# Effectiveness of High‐Intensity Small‐Sided Soccer Games Versus Traditional Soccer Training on Cardiovascular and Metabolic Health Outcomes in Adolescents With Increased Metabolic Risk: A Randomized Controlled Trial

**DOI:** 10.1155/tsm2/1078783

**Published:** 2026-05-13

**Authors:** Nicolás Gómez-Álvarez, Felipe Hermosilla-Palma, Tomás Reyes-Amigo, Mauricio Díaz-Alvarado, Cristian Luarte-Rocha, Juan Pablo Espinoza-Puelles, Rafael Zapata-Lamana, Carlos Cristi-Montero, José Oliveira, Hélder Fonseca

**Affiliations:** ^1^ Centre for Research, Education, Innovation and Intervention in Sport, Faculty of Sport of the University of Porto, Porto, 4200-450, Portugal; ^2^ Centro Regional de Estudios Avanzados en Estilos de Vida Activos y Saludables, Universidad Adventista de Chile, Chillán, Chile, unach.cl; ^3^ Pedagogía en Educación Física, Facultad de Educación, Universidad Autónoma de Chile, Talca, Chile, uautonoma.cl; ^4^ Physical Activity Sciences Observatory (OCAF), Department of Physical Activity Sciences, Universidad de Playa Ancha, Valparaíso, Chile, upla.cl; ^5^ Departamento de Educación y Humanidades, Universidad de Magallanes, Punta Arenas, Chile, umag.cl; ^6^ Facultad de Odontología y Ciencias de la Rehabilitación, Universidad de San Sebastián, Concepción, 4080871, Chile; ^7^ IRyS Group, Physical Education School, Pontificia Universidad Católica de Valparaíso, Valparaíso, Chile, ucv.cl; ^8^ Escuela de Kinesiología, Facultad de Salud, Universidad Santo Tomás, Los Ángeles, 4441171, Chile, ust.cl; ^9^ Escuela de Educación, Universidad de Concepción, Los Ángeles, 4451032, Chile, udec.cl; ^10^ Center for Interdisciplinary Research in Biomedicine, Biotechnology and Well-Being (CID3B), Pontificia Universidad Católica de Valparaíso, Valparaíso, Chile, ucv.cl; ^11^ Research Centre in Physical Activity, Health, and Leisure (CIAFEL), Faculty of Sport, University of Porto, Porto, 4200-450, Portugal, up.pt; ^12^ Laboratory for Integrative and Translational Research in Population Health (ITR), Porto, Portugal

**Keywords:** exercise, lipid profile, metabolic syndrome, obesity, sports

## Abstract

Physical exercise is an important component in obesity and cardiometabolic risk management in adolescents, but improving its effectiveness remains challenging. This study aimed to compare the effectiveness of high‐intensity small‐sided soccer games (SSSGs) with traditional soccer group (TSG) on physical fitness, body composition, blood pressure, and metabolic risk biomarkers in adolescent boys with increased metabolic risk. Fifty‐one boys (11–15 years) with abdominal obesity were recruited and randomly assigned into one of three groups: i) control group (CG; no exercise, *n* = 17), ii) SSSG (*n* = 17), and iii) TSG (*n* = 17). Over 16 weeks, the SSSG and TSG groups engaged in 3 d/week, 60‐min exercise sessions, while the CG continued their usual activities without a structured exercise intervention. SSSG consisted of high‐intensity soccer games, while the TSG mimicked the usual soccer practices. Assessments for body composition, blood pressure, physical fitness, and metabolic risk biomarkers were conducted at baseline and immediately after the intervention. The results showed that compared to the CG, SSSG significantly decreased fat mass (*p* = 0.03) and increased fat‐free mass (*p* = 0.05), while TSG led to significant increases in fat‐free mass (*p* < 0.001) and HbA1c (*p* < 0.01). When comparing both exercise groups, SSSG was shown to be superior in improving fasting insulin (*p* = 0.03), HOMA‐IR (*p* = 0.03), total cholesterol (*p* = 0.04), VLDL cholesterol (*p* = 0.02), triglycerides (*p* = 0.03), and the cardiometabolic risk index SPISE (*p* = 0.02). In conclusion, following a 16‐week intervention, SSSG was shown to be superior to TSG in improving cardiometabolic health compared to TSG in adolescents with increased metabolic risk.

**Trial Registration:** Clinical trial.gov.identifier: NCT06377137

## 1. Introduction

Obesity is a major risk factor for cardiovascular and metabolic diseases, and its prevalence continues to increase worldwide [1]. It is estimated that obesity and its interaction with other metabolic risk factors will continue to significantly contribute to the global burden of morbidity and mortality by 2050 [2]. During early adolescence, physiological, behavioral, and emotional changes occur, favoring excessive body weight increase and, consequently, cardiovascular and metabolic disease risk [3, 4]. As a chronic relapsing disease, long‐term obesity remission is difficult to achieve; therefore, 90% of adolescents living with obesity will become overweight or obese [5]. Hence, practical and feasible interventions must be optimized for the primary prevention and treatment of obesity and metabolic syndrome (MetS) [6].

Increasing energy expenditure through physical exercise programs is an important strategy for preventing and treating obesity [3, 4], especially for improving cardiovascular and metabolic health in patients with obesity [7, 8]. Although the effectiveness of exercise in reducing excess weight is limited [9], exercise may induce greater relative improvements in adipose tissue function [10], secretory profile [11], blood lipids, blood pressure, liver steatosis, and insulin resistance [7, 12, 13]. However, exercise benefits are intrinsically dependent on the correct manipulation of key variables such as mode, volume, and intensity [7, 12–14]. Additionally, long‐term adherence is a critical aspect of success [15]. Therefore, developing scalable, engaging exercise formats applicable in real‐world settings remains a key challenge [16].

Sports participation has been incorporated into public health policies worldwide as a strategy to promote health [17]. Soccer is one of the most widely practiced sports worldwide and combines intermittent high‐intensity actions with rapid directional changes [18–20]. These characteristics are associated with favorable cardiovascular and metabolic adaptations [18, 20], and soccer‐based interventions have demonstrated feasibility in clinical populations [20–22] and are easily scalable [16, 23], making it an optimal strategy for obesity prevention and treatment.

In recent years, small‐sided soccer games (SSSG) have been proposed as a potentially more effective format to structure soccer sessions for promoting cardiometabolic health [18, 20, 24]. SSSG use simplified rules and reduced pitch dimensions to regulate individual participation and physical demands [19, 25]. Compared with recreational traditional soccer training, which typically includes prolonged instructional phases and lower individual engagement, SSSG increase the density of high‐intensity actions and accumulated physiological demands [19, 20, 25]. Despite these higher intensities, perceived exertion is typically moderate and enjoyment high, a combination that may enhance adherence and increase the effective training dose [25, 26]. Repeated exposure to intermittent high‐intensity efforts represents a key stimulus for cardiovascular and metabolic adaptations [18, 20]. However, evidence regarding the effectiveness of SSSG for obesity management, particularly in adolescents with elevated metabolic risk, remains limited and inconsistent [24].

The hypothesis of this study was that high‐intensity SSSG would induce greater improvements in cardiovascular fitness and cardiometabolic risk markers than traditional soccer training, due to greater exposure to high‐intensity efforts and accumulated neuromuscular and metabolic demands. To test this, we performed a randomized controlled trial (RCT) comparing the effects of high‐intensity SSSG with traditional soccer training on surrogates of health‐related fitness, namely, body composition, arterial blood pressure, and biochemical metabolic risk markers, in male adolescents with increased metabolic risk. Clarifying whether structural modifications within soccer training can enhance cardiometabolic adaptations may contribute to optimizing scalable and context‐appropriate health‐promotion strategies for this vulnerable population.

## 2. Material and Methods

### 2.1. Study Design and Participants

This RCT with three arms, two exercise groups, and one control group (CG) was conducted on male adolescents aged 11–15 years with abdominal obesity and adhered to the CONSORT reporting guidelines.

The protocol was approved by the Ethics Committees of the Faculty of Sports of the University of Porto (CEFADE 08‐21) and the Adventist University of Chile (N° 2023‐03) and was conducted according to the principles of the Declaration of Helsinki. Recruitment was performed between March and April 2023 at four schools in Chillán, Chile.

The inclusion criteria were as follows: (i) males with 11–15 years at the start of the intervention, (ii) abdominal obesity ≥ 90^th^ percentile as assessed by WC [27] or WtHR ≥ 0.50 [28], and (iii) available to participate in the study regardless of possible group allocation. The exclusion criteria were as follows: (i) any health condition incompatible with vigorous physical exercise (e.g., cardiac disease, orthopedic or neurological limitations, severe respiratory diseases); (ii) history of significant musculoskeletal injuries in the previous 6 months (e.g., fractures, muscle injuries, or sprains); and (iii) concurrent participation in a structured weight loss or exercise program apart from regular school physical education classes.

Based on the teachers’ anthropometric measurements at the beginning of each school year, legal guardians of potentially eligible participants were contacted with an invitation to participate in the study. Detailed characteristics of the study were provided to participants and legal guardians in the invitation letter and were verbally reinforced later. Written informed consent was obtained from the parents or legal guardians, and written assents were obtained from the adolescents prior to participation.

**FIGURE 1:**
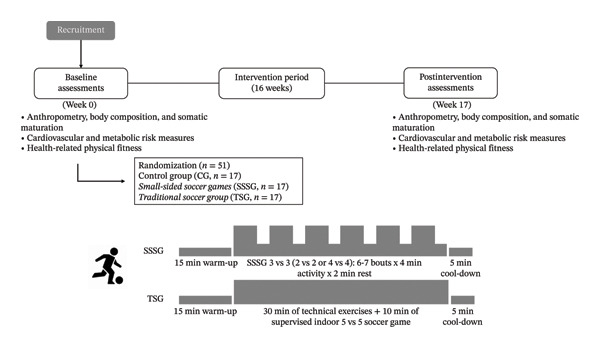
Study design and experimental timeline.

Fifty‐one male adolescents with abdominal obesity met the eligibility criteria and provided their informed assents. A flowchart detailing participant enrollment, allocation, follow‐up, and analysis is shown in Figure 1. After the baseline assessments, the participants were randomly assigned to one of three groups by simple randomization: (i) CG (no exercise), (ii) SSSG, and (iii) traditional soccer group (TSG). Measurements were conducted 1 week before the start of the intervention and 1 week after its completion. Participants were instructed to maintain their usual daily routines throughout the study period, with periodic reminders provided by the research team (Figure 1).

### 2.2. Group Characterization

#### 2.2.1. CG

Participants assigned to the CG did not participate in any weight‐reduction intervention (nutrition, pharmacological, or structured exercise) and were asked to maintain their normal daily activities throughout the study. Compliance was reinforced through periodic reminders from the research team and follow‐up communication with participants and their parents.

For ethical reasons and to encourage participation in postintervention assessments, CG participants were invited to participate in a recreational soccer program led by the research project team at the end of the project.

#### 2.2.2. Exercise Groups

Participants assigned to the SSSG and TSG were scheduled to participate in 48 supervised training sessions (16 weeks), three sessions/week of ∼60 min/session. The 16‐week duration was chosen based on previous RCTs in adolescents with overweight or cardiometabolic risk demonstrating significant improvements after 12 weeks of structured exercise [24], allowing sufficient exposure to induce physiological adaptations within the school term. Each session started with a standard 15‐min warm‐up that included joint movements, linear running, running with directional changes, and jumping, preferably with a ball. The main part of the session lasted ∼ 40 min, and its components are described in Table 1. Each session finished with a 5‐min cool‐down that included low‐intensity aerobic exercises and stretching. Exercise intensity was monitored in all sessions with heart rate monitors (Polar Verity Sense, Polar Electro Oy Inc.).

**TABLE 1:** Characteristics of the main activities training protocol for the small‐sided soccer games group and the traditional soccer group.

SSSG		TSG	
Variables	Description	Variables	Description
Training regimen	Fractioned: 6–7 bouts *x* 4‐min activity *x* 2‐min rest	Technical skills	10 min of instructed dribbling activities with the ball
Format	3v3 (primary format); adjusted to 2v2 or 4v4 according to session attendance		10 min of instructed dribbling and shooting activities
Pitch size	Pitch dimensions were adjusted to maintain ∼27 m^2^ per player (3*vs*3: 9 × 18m; 2*vs*2: 7.5 × 14.5 m; 4*vs*4: 10.5 × 21 m)		10 min of instructed passing and shooting activities
Other adjustments	Strict man‐to‐man marking throughout play	Soccer games	Supervised an indoor 5 *vs* 5 soccer game.
	After scoring, the scoring team retreated to midfield		
	Balls immediately available to reduce game interruptions		
	Coach provided verbal encouragement to maintain high intensity		

Abbreviations: SSSG = small‐sided soccer games, TSG = traditional soccer group.

All sessions were conducted on an indoor futsal court with a polyurethane synthetic sports surface between 16:00 and 18:00 h, from April to August (autumn–winter season in Chile). The facility was an indoor gymnasium without active climate control. Based on climatological records from the Chilean Meteorological Direction, mean outdoor temperatures in Chillán during the intervention period ranged from approximately 20°C–11°C during the intervention hours, with relative humidity ranging between 52% and 70%.

#### 2.2.3. SSSG

SSSG‐based training was designed to generate physiological responses following a high‐intensity interval training concept (Table 1). Based on a pilot study, we estimated that participants would be at vigorous intensity for approximately 80% of the time (> 77% maximum heart rate) during each SSSG [26]. SSSG design was based on previous studies, reporting that a small number of players, reduced pitch size, man‐marking task assignment, and frequent verbal encouragement from the trainer were associated with higher intensity [25, 26].

To maintain comparable spatial constraints across formats, pitch dimensions were adjusted to provide an approximate relative playing area of ∼27 m^2^ per player. This value was selected based on previous evidence, indicating that smaller relative areas per player are associated with higher physiological demands, likely due to increased involvement in play, greater frequency of technical actions, and reduced recovery opportunities [25]. Additionally, pilot testing confirmed that this configuration allowed participants to consistently reach vigorous‐intensity exercise while preserving game flow and engagement [26].

In addition, rules that favored the game flow were used, such as (i) to score a goal, the entire team had to be in the opponent’s middle field and (ii) balls were immediately available to reduce game interruptions.

#### 2.2.4. TSG

The TSG followed a recreational traditional soccer training format adapted from a previously published protocol [29]. Each session included 30 min of instructor‐led technical drills performed individually or in pairs (e.g., dribbling, shooting, and passing), followed by a 10‐min match organized according to the original characteristics of an indoor soccer game, without specific task constraints to manipulate intensity (Table 1).

### 2.3. Measurements and Outcomes

All participants were assessed 1 week before the start of the intervention and 1 week after its completion to determine anthropometry, body composition, and cardiometabolic risk profiles. Waist circumference and cardiorespiratory fitness (CRF) were defined as the study’s co‐primary outcomes. Other measures of cardiometabolic risk and physical fitness were the secondary outcomes.

### 2.4. Anthropometry, Body Composition, and Somatic Maturation

Assessments were conducted between 9:00 a.m. and 12:00 a.m. Participants were instructed not to engage in vigorous physical activity for at least 24 h and not to eat for at least 2 hours before the assessments. While wearing only light clothing, body mass (anthropometric scale model 813; Seca, Hamburg, Germany), height, sitting height (anthropometric stadiometer model 213; Seca), and WC (anthropometric measuring tape, model 201 Seca) were measured using standard procedures. Cutoff points based on BMI z‐scores were used to define overweight (>+1 SD) and obesity (> + 2 SD) [30]. For abdominal obesity, age and sex‐specific percentiles were used to classify WC as being at risk for abdominal obesity (≥ *p*75 *y* < *p*90) or established abdominal obesity (≥ *p*90) [27]. Regarding WtHR, a cutoff point of 0.5 was used to classify participants at cardiometabolic risk [28]. Body composition was measured using an InBody 270 multifrequency analyzer (InBody 270, Biospace, California, USA) [31]. The muscle mass (kg), body fat (kg and %), and fat‐free mass (kg) were calculated using the manufacturer’s algorithm.

Somatic maturation was assessed by determining peak height velocity (PHV) using the formula described by Mirwald et al. [32] = − 9.236 + (0.0002708 ∗ (leg length ∗ sitting height)) + (−0.001663 ∗ (age ∗ leg length)) + (0.007216 (age ∗ sitting height)) + (0.02292 ∗ (mass by stature ratio ∗ 100)).

### 2.5. Cardiovascular and Metabolic Risk Measures

Systolic blood pressure (SBP) and diastolic blood pressure (DBP) were measured twice in the right arm using an automatic sphygmomanometer (Omron HEM 7130) after 5 min of rest, following the recommendations of the National High Blood Pressure Education Working Group [33]. The average of both measurements was used to classify BP according to age‐ and height‐specific percentiles as normal (50th percentile), elevated BP (> 90th percentile), Stage 1 hypertension (≥ 95th percentile), or Stage 2 hypertension (≥ 95th percentile + 12 mmHg) [33]. The lowest resting heart rate was also recorded during blood pressure measurements as a surrogate for resting BP.

Venous blood samples were obtained from the antecubital vein between 8:00 and 10:00 a.m. after a 10‐ to 12‐h overnight fast. The samples were handled according to the standard procedures in a private clinical pathology laboratory. The following biochemical parameters were obtained: serum levels of lipid profile (high‐density lipoprotein cholesterol [HDL‐C], low‐density lipoprotein cholesterol [LDL‐C], very low‐density lipoprotein cholesterol [VLDL‐C], total cholesterol, and triglycerides [TG]), liver enzymes (alanine aminotransferase [ALT], aspartate aminotransferase [AST], and *γ*‐glutamyl transpeptidase [GGT]), and glucose metabolism (insulin, fasting glucose, homeostatic model assessment for insulin resistance [HOMA‐IR], and glycated hemoglobin A1c [HbA1c]). The single‐point insulin sensitivity estimator (SPISE) was calculated as a surrogate of MetS risk [SPISE = 600∗ HDL^0.185/(TG^0.2∗ BMI^1.338)] [34]. SPISE has been shown to have high sensitivity (90.4%) and specificity (76.1%) for detecting MetS in Chilean children and adolescents [34]. An SPISE cutoff value ≤ 5.4 was used to determine a high risk of MetS and insulin resistance [34]. Based on the recommendations of the National Heart Lung and Blood Institute and replicated for Chilean children and adolescents, participants’ risk of dyslipidemia was classified as follows: (i) without risk (TC < 170 mg/dL; TG < 90 mg/dL; LDL‐C < 110 mg/dL; HDL‐C > 45 mg/dL), (ii) at‐risk (TC ≥ 170 < 200 mg/dL; TG ≥ 90 < 130 mg/dL; LDL‐C ≥ 110 < 130 mg/dL; HDL‐C ≥ 40 ≤ 45 mg/dL), or iii) at high‐risk (TC ≥ 200 mg/dL, TG ≥ 130 mg/dL, LDL‐C ≥ 130 mg/dL, HDL‐C < 40 mg/dL) [35].

### 2.6. Health‐Related Physical Fitness

CRF was assessed using the 1‐mile run/walk test [36]. The participants were motivated to complete the 1‐mile (1609 m) distance in the shortest possible time. Time in minutes was considered the test result. No formal familiarization session was conducted; however, these tests are routinely implemented within the school’s physical education curriculum, and participants were familiar with their procedures.

Handgrip strength (HGS) was assessed as a surrogate of upper limb strength with a digital dynamometer (JAMAR Plus Digital Hand Dynamometer, Illinois, United States) [37]. Before the recorded trials, participants performed three practice attempts per hand to ensure correct technique and understanding of the procedure. Subsequently, two trials per hand were recorded in a standing position with the arm extended parallel to the body. The highest value for each hand was summed and recorded as the HGS (kg).

Lower limb strength was assessed using the horizontal jump test [37]. The test was performed three times, and the longest jump, recorded in cm, was used as the test result.

The order of test administration was HGS, horizontal jump, and finally the 1‐mile run/walk test.

### 2.7. Statistical Analysis

Sample size was calculated a priori using G∗power (Version 3.1.9.6) to detect a significant difference between groups on WC and CRF with a magnitude of Cohen’s *d* = 0.25 [21], assuming a two‐sided type one error probability of 5% and a statistical power of 80%. Based on these assumptions, the recommended total sample size was 42.

A primary analysis was performed to compare the results between the groups as randomized (intention‐to‐treat approach). A secondary analysis was also performed, dividing the exercise intervention groups (SSSG and TSG) into two subgroups according to intervention adherence (attendance rate to exercise sessions < 50% *vs.* ≥ 50%), which were then independently compared to the CG. This analysis was performed to determine whether the effects of exercise intervention on selected outcomes differed according to attendance rate (per protocol analysis).

Primary and secondary analyses were performed using generalized linear mixed models (GLMM) with a gamma distribution and logarithmic link function. Marginal means were estimated, multiple post hoc comparisons were performed for the different groups and times, and statistical tests were adjusted using the Bonferroni method. The effect size of the differences between each group was expressed as Cohen’s *d* with a 95% confidence interval. Effect sizes were classified as trivial (< 0.2), small (0.2‐0.6), moderate (0.6–1.2), or large [38].

For the primary analyses, the model considered fixed factors for the group (CG, SSSG, and TSG) and time (baseline and post‐16 weeks), whereas the random factors considered subjects. The secondary analysis followed the same principle, in which the group factor included five levels (CG, SSSG < 50%, SSSG ≥ 50%, TSG < 50%, and TSG ≥ 50%) and time (baseline and post‐16 weeks). Baseline continuous values of the dependent variable and somatic maturation (PHV) were included as adjustment covariates in the model. The treatment effect was tested based on the differences between the intervention and CGs, using the ratio of the estimated means with a 95% confidence interval.

To identify changes in categorical variables over time, the McNemar test was used, considering only participants with complete data at baseline and after 16 weeks of intervention. Owing to the number of participants per category, we created only two categories: at‐risk or nonrisk for blood pressure and lipid profile measurements.

All statistical procedures were performed using *R*, Version 4.0.0 (R Project for Statistical Computing). The threshold for statistical significance was *p* < 0.05.

## 3. Results

**FIGURE 2:**
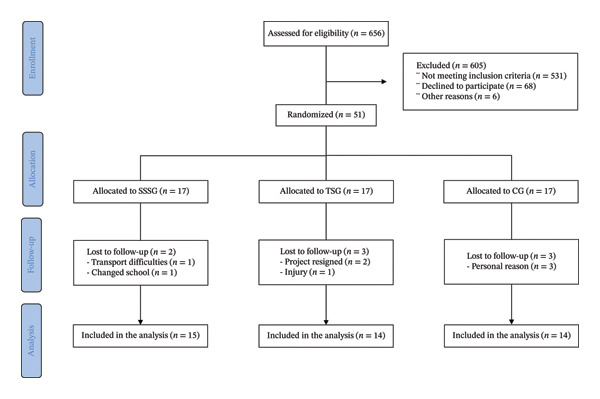
Flowchart of participant enrollment, group allocation, follow‐up, and final analysis.

One hundred and twenty‐five adolescents were invited to participate in the study; however, 18 did not meet the inclusion criteria, 50 did not accept participation, and six did not respond to the invitation (Figure 2). Finally, 51 participants were included in the final sample and randomized to the SSSG (*n* = 17), TSG (*n* = 17), or CG (*n* = 17). The mean ± standard deviation (SD) age was 13.14 ± 1.42 years, weight was 76.41 ± 15.36 kg, BMI z‐score was 2.59 ± 0.47, WC was 92.99 ± 8.97 cm, and PHV was −1.04 ± 1.29 years. At baseline, 80.3% had abdominal obesity, 19.6% were at risk for abdominal obesity, 2% had elevated blood pressure, 6% had Stage 1 hypertension, 6% were at risk for dyslipidemia, 22% had elevated TC (2% at risk and 20% high risk), 54% had low HDL‐C (29% at risk and 25% high risk), 12% had elevated LDL‐C (8% at risk and 4% high risk), and 53% had elevated TG (31% at risk and 22% high risk). None of the participants had high fasting blood glucose levels at baseline. No significant differences were identified between the groups at baseline in anthropometry, body composition, cardiovascular and metabolic risk variables, or physical fitness.

Eight participants dropped out of the study: two in the SSSG, three in the TSG, and three in the CG. Overall, the age (CG, 12.30 ± 0.95 years; SSSG, 13.95 ± 0.21 years; TSG, 13.23 ± 2.12 years), BMI z‐score (CG, 2.34 ± 0.60; SSSG, 2.79 ± 0.27; TSG, 2.18 ± 0.25), WC (CG, 89 ± 14.80 cm; SSSG, 95.35 ± 3.32 cm; TSG, 88.83 ± 4.25 cm), and CRF (CG, 14.70 ± 1.63 min; SSSG, 12.72 ± 0.35 min; TSG, 13.47 ± 2.62 min) of participants who dropped out of the study did not differ from the characteristics of participants who completed assessments at baseline and at the end of the intervention. No significant differences were identified in the attendance rate of the SSSG and TSG sessions (42.5 ± 23.8% vs 43.0 ± 21.9%, *p* = 0.96). Regarding exercise session average intensity according to HR response, SSSG sessions were more intense than TSG (average %HRmax, 81.1 ± 9.5% vs 76.7 ± 10.5%; *p* < 0.001). Specifically, participants in the SSSG group spent significantly more time in the 80%–90% HRmax zone (32.2 ± 11.6% vs 24.7 ± 11.9%, *p* < 0.001) and above 90% HRmax (24.5 ± 19.6% vs 15.3 ± 15.6%, *p* < 0.001). In contrast, the TSG group spent more time in the 70%–80% HRmax zone (28.9 ± 10.0% vs 26.0 ± 9.9%, *p* = 0.02).

### 3.1. Intention‐to‐Treat Analysis

Table 2 shows the results at baseline and after intervention in each group. Regarding the primary outcomes of the study (WC and 1‐mile walk/run test), no significant differences were identified between baseline and after 16 weeks of intervention in SSSG (WC ratio: 1.03 [95% CI: 0.99–1.06]; *p* = 0.16; 1‐mile walk/run test ratio: 1.07 [95% CI: 0.99–1.15]; *p* = 0.23), TSG (WC ratio: 1.01 [95% CI; 0.97–1.04]; *p* = 1.00; 1‐mile walk/run test ratio: 1.08 [95% CI: 0.99–1.17]; *p* = 0.08), or CG (WC ratio: 1.01 [95% CI: 0.97–1.04]; *p* = 1.00; 1‐mile walk/run test ratio: 1.01 [95% CI: 0.93–1.10]; *p* = 0.45) (Figure 3). Similarly, no significant differences in the co‐primary outcomes were identified for comparisons between the CG and both exercise groups (CG vs. SSSG or CG vs. TSG, *p* > 0.05) or between exercise groups (SSSG vs. TSG, *p* > 0.05). The between‐group comparisons of all variables are presented in Supporting Table 1.

**TABLE 2:** Effects of SSSG and TSG on anthropometry, body composition, cardiometabolic risk factors, and health‐related physical fitness.

	CG			SSSG			TSG		
	Baseline	Postintervention	*p*‐value	Baseline	Postintervention	*p*‐value	Baseline	Postintervention	*p*‐value
*Anthropometry and body composition*									
Weight (kg)	75.2 (73.5–76.9)	75.3 (73.5–77.2)	1.00	75.5 (73.8–77.3)	75.1 (73.3–76.9)	1.00	76.0 (74.3–77.8)	76.4 (74.5–78.3)	1.00
Height (cm)	162.7 (162.3–163.1)	164.5 (164.1–165.0)	**< 0.001**	162.5 (162.1–162.9)	164.9 (164.5–165.3)	**< 0.001**	162.6 (162.2–163.0)	165.0 (164.5–165)	**< 0.001**
BMI (kg/m2)	28.5 (28.0–29.0)	27.8 (27.3–28.4)	0.28	28.6 (28.1–29.1)	27.7 (27.2–28.2)	0.02	28.4 (27.9–28.9)	28.2 (27.6–28.7)	1.00
BMI Z‐score	2.56 (2.48–2.66)	2.41 (2.32–2.51)	**0.02**	2.59 (2.50–2.68)	2.40 (2.31–2.49)	**< 0.001**	2.53 (2.44–2.62)	2.42 (2.33–2.52)	0.30
WC (cm)	92.8 (90.9–94.7)	92.3 (90.3–94.3)	1.00	93.0 (91.1–94.9)	90.4 (88.5–92.3)	0.16	92.6 (90.8–94.4)	92.1 (90.2–94.1)	1.00
WtHR	0.57 (0.56–0.58)	0.56 (0.55–0.58)	1.00	0.57 (0.56–0.59)	0.55 (0.54–0.57)	0.06	0.57 (0.56–0.58)	0.56 (0.54–0.57)	0.62
Fat mass (kg)	28.2 (26.6–30.0)	29.8 (27.9–31.8)	1.00	28.6 (26.9–30.4)	27.6 (25.9–29.4)	1.00	27.1 (25.5–28.8)	27.8 (26.0–29.7)	1.00
Fat mass (%)	37.3 (36.1–38.5)	37.9 (36.6 to 39.2)**^c^**	1.00	37.2 (36.0–38.4)	36.1 (34.9 to 37.3)**^a^**	0.24	36.6 (35.4–37.8)	36.7 (35.5–37.9)	1.00
Fat‐free mass (kg)	47.1 (45.9–48.4)	46.0 (44.8 to 47.3)**^b^** ^**,**^ **^c^**	0.05	46.6 (45.3–47.9)	47.1 (45.8 to 48.4)**^a^**	1.00	46.5 (45.2–47.7)	47.9 (46.6 to 49.2)**^a^**	**< 0.01**
Muscle mass (kg)	26.0 (25.2–26.9)	26.1 (25.2–27.0)	1.00	25.7 (24.8–26.6)	25.9 (25.0–26.9)	1.00	25.7 (24.8–26.5)	26.2 (25.3–27.1)	0.89

*Cardiovascular and metabolic risk factors*									
Rest heart rate (bpm)	76.87 (73.46–80.45)	75.36 (71.72–79.18)	1.00	77.21 (73.77–80.81)	71.22 (67.86–74.75)	**0.04**	76.12 (72.74–79.66)	72.41 (68.90–76.10)	0.99
SBP (mmHg)	110.47 (107.29–113.74)	105.01 (101.71–108.41)	0.07	111.22 (108.00–114.52)	107.41 (104.15–110.78)	0.69	111.04 (107.86–114.32)	110.62 (107.16–114.18)	1.00
DBP (mmHg)	66.48 (64.07–68.98)	64.13 (61.56–66.81)	1.00	66.55 (64.24–68.93)	62.84 (60.39–65.38)	0.09	67.06 (64.63–69.58)	64.17 (61.65–66.80)	0.74
Insulin	14.87 (12.38–17.87)	13.77 (11.38–16.67)	1.00	15.17 (12.72–18.09)	12.30 (10.11 to 14.96)**^c^**	0.90	14.07 (11.83–16.74)	**17.16 (14.15**–20.81)**^b^**	1.00
HOMA‐IR	3.28 (2.69–4.00)	3.12 (2.54–3.84)	1.00	3.37 (2.79–4.07)	2.76 (2.23 to 3.41)**^c^**	1.00	3.12 (2.58–3.76)	3.94 (3.20 to 4.86)**^b^**	0.75
HbA1c (%)	5.01 (4.97–5.06)	5.07 (5.02–5.12)	1.00	5.02 (4.98–5.07)	5.11 (5.06–5.16)	0.06	5.02 (4.98–5.07)	**5.21 (5.15**–5.26)**^a^**	**< 0.001**
Glucose (mg/dL)	89.18 (86.93–91.48)	91.33 (88.96–93.78)	1.00	89.59 (87.46–91.78)	89.20 (86.88–91.57)	1.00	88.79 (86.67–90.95)	92.66 (90.25–95.14)	0.06
TC (mg/dL)	151.96 (145.91–158.27)	134.84 (129.31–140.61)	**< 0.001**	150.03 (144.43–155.85)	**132.33 (126.98**–137.92)**^c^**	**< 0.001**	149.88 (144.27–155.70)	**142.01 (136.27**–148.00)**^b^**	0.15
HDL‐C (mg/dL)	45.25 (43.06–47.55)	42.42 (40.33–44.63)	0.14	45.06 (43.03–47.19)	43.36 (41.25–45.57)	1.00	45.28 (43.25–47.41)	45.74 (43.53–48.06)	1.00
LDL‐C (mg/dL)	84.52 (78.66–90.81)	74.00 (68.73–79.67)	**< 0.01**	81.92 (76.60–87.61)	71.15 (66.19–76.47)	**< 0.001**	80.75 (75.51–86.35)	73.98 (68.82–79.52)	0.14
VLDL‐C (mg/dL)	19.51 (16.87–22.56)	15.72 (13.50–18.31)	0.06	19.92 (17.40–22.81)	14.75 (12.73 to 17.10)**^c^**	**< 0.001**	19.00 (16.56–21.81)	18.51 (15.97 to 21.46)**^b^**	1.00
TG (mg/dL)	96.97 (83.91–112.08)	78.93 (67.82–91.86)	0.10	99.56 (87.00–113.94)	74.03 (63.91 to 85.76)**^c^**	**< 0.001**	94.46 (82.33–108.37)	91.88 (79.31 to 106.45)**^b^**	1.00
GGT (U/L)	20.76 (18.70–23.05)	16.05 (14.38–17.91)	**< 0.01**	21.17 (19.18–23.36)	16.60 (14.89–18.51)	**< 0.001**	20.13 (18.25–22.21)	16.18 (14.50–18.05)	0.02
ALT (U/L)	19.41 (16.38–23.01)	23.02 (19.32–27.44)	0.82	18.48 (15.75–21.68)	22.01 (18.50–26.19)	0.69	18.25 (15.56–21.41)	20.40 (17.12–24.30)	1.00
AST (U/L)	21.54 (19.80–23.44)	23.66 (21.68–25.81)	0.57	21.20 (19.56–22.97)	23.86 (21.87–26.02)	0.12	21.33 (19.68–23.11)	24.69 (22.63–26.94)	**0.02**
AST/ALT ratio	1.14 (1.03–1.25)	1.06 (0.96–1.17)	1.00	1.17 (1.07–1.28)	1.13 (1.02–1.24)	1.00	1.16 (1.06–1.27)	1.25 (1.13–1.37)	1.00
SPISE (UA)	5.47 (5.21–5.75)	5.85 (5.55–6.17)	0.11	5.46 (5.21–5.73)	6.11 (5.81 to 6.43)**^c^**	**< 0.001**	5.43 (5.18–5.69)	5.54 (5.27 to 5.82)**^b^**	1.00

*Health-related physical fitness*									
1‐mile run/walk test (min)	13.3 (12.7–14.0)	13.2 (12.5–13.9)	1.00	13.3 (12.7–13.9)	12.4 (11.8–13.1)	0.23	13.1 (12.5–13.8)	12.2 (11.6–12.8)	0.08
HGS (kg)	56.0 (52.9–59.2)	56.8 (53.6–60.2)	1.00	57.7 (54.6–61.1)	59.7 (56.4–63.3)	0.27	56.0 (52.9–59.3)	57.9 (54.6–61.4)	0.34
HJ (cm)	128 (122–134)	128 (122–135)	1.00	128 (123–134)	136 (129–142)	0.24	127 (122–133)	130 (124–136)	1.00

*Note:* CRF, cardiorespiratory fitness; HGS, handgrip strength; HOMA‐IR, homeostatic model assessment for insulin resistance; HbA1c, glycated hemoglobin A1c, TC, total cholesterol; HDL‐C, high‐density lipoprotein cholesterol; LDL‐C, low‐density lipoprotein cholesterol; VLDL‐C, very low‐density lipoprotein cholesterol; TG, triglycerides; ALT, alanine aminotransferase; AST, aspartate aminotransferase; GGT, *γ*‐glutamyl transpeptidase; SPISE, single‐point insulin sensitivity estimator. Bold postintervention values indicate significant between‐group differences identified in post hoc comparisons. Bold *p*‐values indicate statistically significant within‐group changes. Superscripts indicate the group differences. Data: Estimated mean (confidence interval 95%). Models adjusted for baseline values of the dependent variable and peak height velocity.

Abbreviations: BMI = body mass index, CG = control group, DBP = diastolic blood pressure, HJ = horizontal jump, SBP = systolic blood pressure, SSSG = small‐sided soccer games group, TSG = traditional soccer group, WC = waist circumference, WtHR = waist‐to‐height ratio.

^a^different from CG.

^b^different from SSSG.

^c^different from TSG.

FIGURE 3Results for the primary outcomes by group at baseline and after 16 weeks of intervention. (a) Results for cardiorespiratory fitness (1‐mile run/walk test); (b) results for waist circumference. Data are shown as estimated mean and 95% CI. Models adjusted for baseline values of the dependent variable and peak height velocity.

**Figure:**
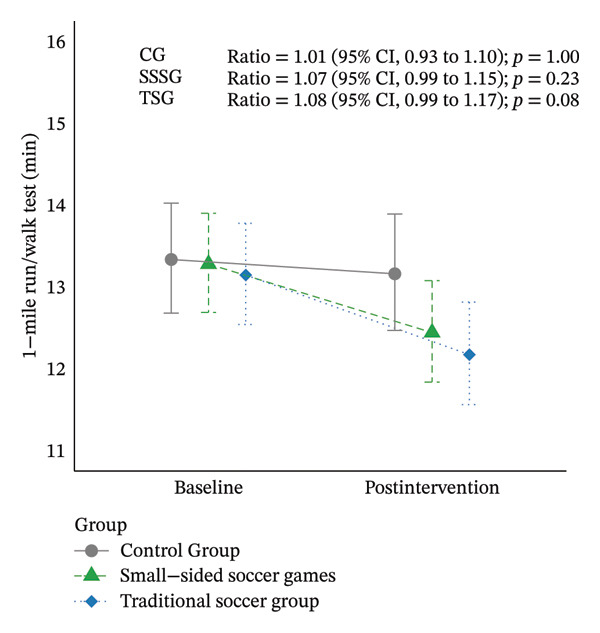
(a)

**Figure:**
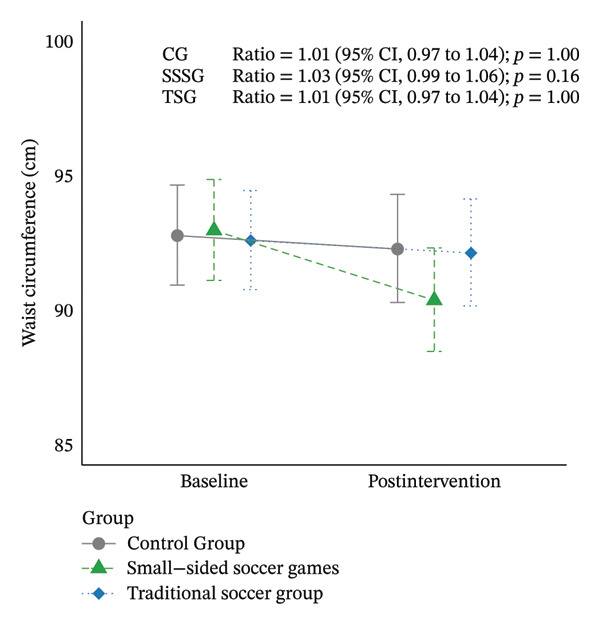
(b)

Significant differences were found between the baseline and post‐16 weeks of intervention in secondary outcomes related to anthropometry, body composition, and cardiometabolic risk. Regarding anthropometric and body composition measures, significant height increases were identified in all groups (*p* < 0.001) and significant decreases in SSSG for BMI (ratio: 1.03 [95% CI: 1.003–1.06]; *p* = 0.02) and BMI z‐score (ratio: 1.08 [95% CI: 1.02–1.06]; *p* < 0.001), while in TSG, increased fat‐free mass was identified (ratio: 0.97 [95% CI: 0.95–0.99]; *p* < 0.001). In addition, significant decreases were recorded in the CG for BMI z‐score (ratio: 1.06 [95% CI: 1.01–1.12]; *p* = 0.02) and fat‐free mass (ratio: 1.02 [95% CI: 1.00–1.05]; *p* < 0.001).

**FIGURE 5:**
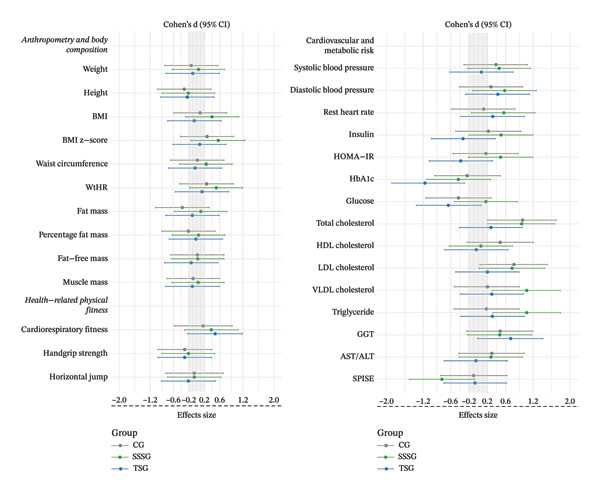
Effect size for comparisons between baseline and after 16 weeks of intervention.

MetS risk, assessed by SPISE, decreased only in the SSSG (ratio: 0.89 [95% CI: 0.83–0.96], *p* < 0.001) (Figure 4). Regarding lipid profile, significant differences between pre‐ and postintervention were identified in the SSSG (TC ratio: 1.13 [95% CI: 1.07–1.21], *p* < 0.0001; LDL ratio: 1.15 [95% CI: 1.04–1.27], *p* < 0.001; VLDL ratio: 1.35 [95% CI: 1.10–1.67], *p* < 0.001; TG ratio: 1.35 [95% CI: 1.09–1.67]; *p* < 0.001), and CG (TC ratio: 1.13 [95% CI: 1.06–1.20], *p* < 0.0001; LDL ratio: 1.14 [95% CI: 1.03–1.26], *p* < 0.0001). Significant changes in glucose metabolism were also identified, but only in TSG with an increase in HbA1c (ratio: 0.96 [95% CI: 0.95–0.98], *p* < 0.0001). Assessment of liver injury markers showed significant reductions in GGT levels in all three groups (CG ratio: 1.29 [95% CI: 1.06–1.58], *p* = 0.002; SSSG ratio: 1.28 [95% CI: 1.05–1.55], *p* = 0.003; TSG ratio: 1.24 [95% CI: 1.02–1.51], *p* < 0.02). Figure 5 shows the effect size for changes in variables assessed between baseline and postintervention according to the group.

FIGURE 4Results on global measure of metabolic syndrome risk according to SPISE score (an increase in SPISE score indicates a lower risk). (a) Estimated means and their confidence intervals at baseline and postintervention per group; (b) percentage of participants per group at risk (SPISE < 5.4) or not at risk of MetS (SPISE ≥ 5.4) at baseline and after 16 weeks of intervention. Note: Figure 4(a), GLMM adjusted for baseline values of the dependent variable and peak height velocity.

**Figure:**
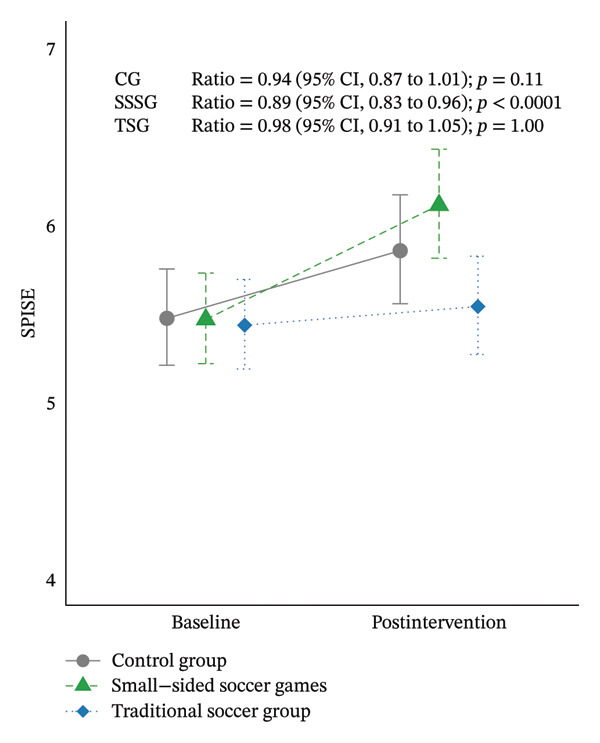
(a)

**Figure:**
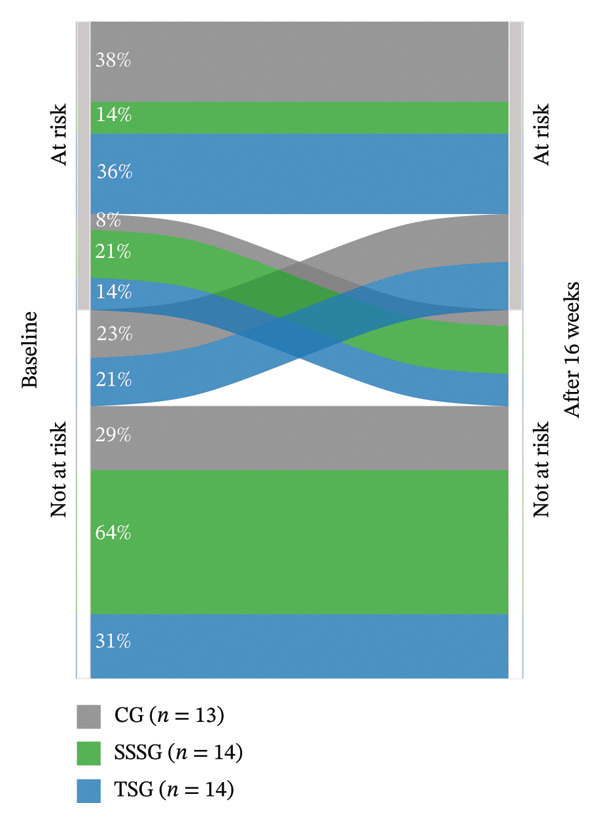
(b)

The McNemar test results showed no significant changes in the proportion of participants at cardiometabolic risk or without cardiometabolic risk after 16 weeks of intervention in either group (*p* > 0.05) (Table 3). However, we observed a tendency for the SSSG group to change from an at‐risk to a no‐risk category. Figure 4(b) shows the SPISE changes, reflecting a higher percentage of SSSG participants (21%) who changed from an at‐risk to not‐at‐risk category compared with participants in the CG (8%) and TSG (14%). Importantly, the number of participants who changed from the not‐at‐risk to at‐risk category was also higher in the CG (23%) and TSG (21%), whereas there was no change in the SSSG.

**TABLE 3:** Number of participants who changed their risk or nonrisk behavior by the end of the intervention.

		**CG**			**SSSG**			**TSG**		
		**Baseline**	**Postintervention**	**p** **-value**	**Baseline**	**Postintervention**	**p** **-value**	**Baseline**	**Postintervention**	**p** **-value**
BMI	Obesity (*n*)	12	11	1.00	15	14	1.00	11	12	1.00
	Overweight (*n*)	2	3		0	1		3	2	

WC	Abdominal obesity (*n*)	11	9	0.63	13	9	0.13	11	9	0.5
	Abdominal obesity (*n*)	3	5		2	6		3	5	

WtHR	Risk (*n*)	14	14	1.00	15	13	0.5	14	11	0.25
	At risk (*n*)	0	0		0	2		0	3	

DBP	No risk (*n*)	14	14	na	15	15	na	14	14	na
SBP	At risk (*n*)	0	1	1.00	2	0	0.5	1	1	1.00
	No risk (*n*)	14	13		13	15		13	13	

TC	At risk (*n*)	1	0	1.00	3	0	0.25	4	3	1.00
	No risk (*n*)	13	14		11	14		10	11	

HDL‐C	At risk (*n*)	8	8	1.00	7	9	0.5	9	8	1.00
	No risk (*n*)	6	6		7	5		5	6	

LDL‐C	At risk (*n*)	0	0	1.00	0	0	1.00	3	1	0.5
	No risk (*n*)	14	14		14	14		11	13	

TG	At risk (*n*)	7	4	0.38	7	2	0.06	7	9	0.62
	No risk (*n*)	7	10		7	12		7	5	

SPISE	At‐risk MetS (*n*)	8	6	0.62	5	2	0.25	8	7	1.00
	No‐risk MetS (*n*)	5	7		9	12		6	7	

*Note:* TG = triglycerides.

Abbreviations: BMI = body mass index, DBP = diastolic blood pressure, HDL‐C = high‐density lipoprotein cholesterol, LDL‐C = low‐density lipoprotein cholesterol, SBP = systolic blood pressure, SPISE = single‐point insulin sensitivity estimator, TC = total cholesterol, WC = waist circumference, WtHR = waist‐to‐height ratio.

### 3.2. Per‐Protocol Analysis

Regarding training attendance, 47% (*n* = 7) and 43% (*n* = 6) of the participants attended less than 50% of the scheduled sessions in the SSSG and TSG, respectively. Attendance analysis and results based on attendance can be found in the supplement.

Analyses of the main outcomes found no significant differences in WC or CRF between baseline and after 16 weeks of intervention or between the groups (*p* > 0.05). However, a trend was observed for a greater reduction in WC in the high‐attendance SSSG group (ratio: 1.04 [95% CI: 0.99–1.10]; *p* = 0.20) than in the low‐attendance SSSG group (ratio: 1. 01 [95% CI: 0.96–1.06]; *p* = 1.00) and to both TSG groups (high attendance TSG ratio: 1.01 [95% CI: 0.96–1.06]; *p* = 1.00; low attendance TSG ratio: 0.99 [95% CI: 0.94–1.05]; *p* = 1.00). Changes in CRF showed similar trends in the low‐attendance (low‐attendance SSSG ratio: 1.08 [95% CI: 0.96–1.22]; *p* = 1.00; low‐attendance TSG ratio: 1.11 [95% CI: 0.97–1.26]; *p* = 0.47) and high‐attendance groups (high‐attendance SSSG ratio: 1.05 [95% CI: 0.93–1.19]; *p* = 1.00; high‐attendance TSG ratio: 1.06 [95% CI: 0.94–1.20]; *p* = 1.00) (Figure 6).

FIGURE 6Results of primary outcomes by group at baseline and after 16 weeks of intervention according to adherence to training (high adherence = > 50%, low attendance < 50% attendance). (a) Results for cardiorespiratory fitness (1‐mile run/walk test); (b) results for waist circumference. Data are shown as estimated mean and 95% CI. GLMM models adjusted for baseline values of the dependent variable and peak height velocity.

**Figure:**
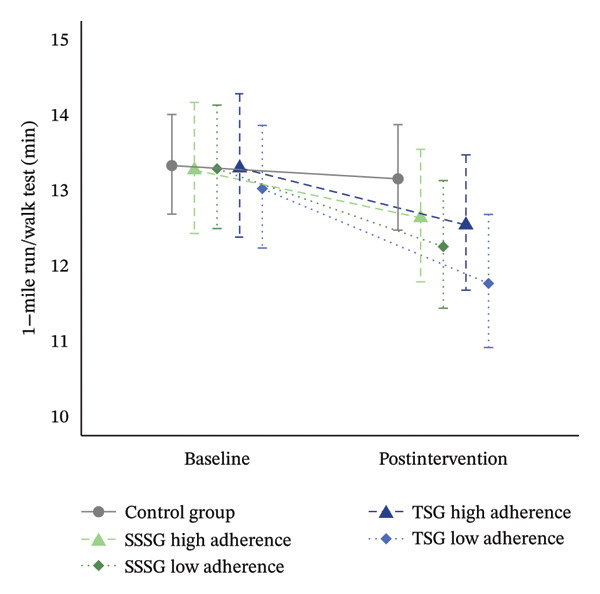
(a)

**Figure:**
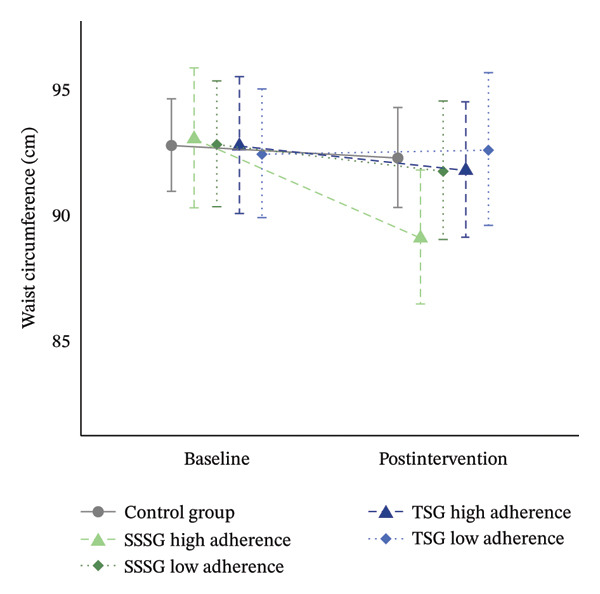
(b)

For the remaining variables, although trends toward a greater magnitude of difference between before and after the intervention were identified in participants with higher attendance rates, these were not statistically significant.

## 4. Discussion

The main findings of this study showed that after 16 weeks of intervention, a high‐intensity SSSG induced more favorable changes in anthropometric measures (BMI and BMI z‐score), serum lipid profile (TC, LDL, VLDL, and TG), GGT, and the risk index for MetS SPISE compared to TSG, which only improved fat‐free mass and circulating GGT liver enzyme concentration. Surprisingly, after the intervention, the TSG group showed a significant increase in HbA1c levels. The CG group showed significant decreases in BMI z‐scores and TC, LDL‐C, and GGT levels. Between‐group comparisons showed that SSSG, compared to TSG, induced beneficial changes in a higher number of metabolic risk factors, including HOMA‐IR, HbA1c, TC, VLDL‐C, TG, and the SPISE index. Despite these benefits, our results suggest limited effects of SSSG and TSG compared to CG, with both exercise interventions showing mainly beneficial improvements in body composition, in particular, significant increases in fat‐free mass and, in the case of SSSG, a significant decrease in fat mass percentage.

The limited effects on measures of cardiometabolic risk found in this study between the exercise groups and the CG contrasts with suggestions from previous systematic reviews that recreational soccer promotes substantial improvements in cardiometabolic health and fitness in children and adolescents [18, 20]. However, these conclusions are limited by the lack of meta‐analyses, which could have increased the risk of overestimating the beneficial effects of soccer‐based interventions. For example, although our between‐group results only showed differences in body composition, the intragroup results suggested that SSSG significantly improved cardiovascular and metabolic risk factors. This observation is aligned with previous studies conducted in children and adolescents with overweight or obesity, showing significant pre‐ to postintervention effects but inconsistent findings in comparison with a CG [24]. These results reinforce the need to further investigate how to optimize this type of intervention for obesity and cardiometabolic health improvements in this population.

Interestingly, the results from this study showed that there were more differences between exercise groups (SSSG vs. TSG) than between SSSG and the CG. SSSG showed improvements in HOMA‐IR, HbA1c, TC, VLDL‐C, TG, and SPISE index compared to the TSG. Although some of these findings were surprising, they might be associated with developmental changes that occur during puberty [39]. CG increased height without concomitant changes in weight or body composition. This led to a reduction in BMI z‐scores from pre‐ to postintervention assessment, which, however, may not necessarily reflect a benefit in cardiometabolic health [40]. Although control participants were instructed to maintain their usual lifestyle, dietary intake was not objectively monitored. Therefore, we cannot exclude the possibility that increased awareness of cardiometabolic risk due to study participation may have led to unintended behavioral changes, such as dietary improvements, potentially influencing lipid profile outcomes.

The results suggest that there is a tendency for SSSG to be more effective than TSG in promoting cardiometabolic health improvements. Based on the SPISE index [34], SSSG intervention was the only one that significantly decreased the risk of MetS and the one in which more participants moved from an at‐risk to a no‐risk classification, as well as the only group in which no participant transitioned from a no‐risk to an at‐risk classification. Previous studies have recommended using indices to assess the effect of a training program on cardiometabolic risk [41]. In this study, we used the SPISE index because a cutoff point of 5.4 was shown to effectively predict insulin resistance and MetS independent of age or pubertal status in Chilean children and adolescents [34]. Additionally, it has been suggested that SPISE could be a predictor of nonalcoholic fatty liver disease [42], which is an increasing health concern in children and adolescents [43].

This decrease in MetS risk was associated with consistent changes in BMI and blood lipid profiles, as shown at the end of the SSSG intervention. The differences between pre‐ and postintervention in SSSG were significant, with a moderate effect size on all measures of blood lipid profile except HDL‐C, and significant decreases with a small effect size on BMI, BMI z‐score, and resting heart rate. On the other hand, our results showed no significant effects on surrogates of glucose metabolism or blood pressure, probably because at the study’s baseline, no participants had any glucose metabolism dysfunction, and few had elevated SBP.

The results reported in this study are consistent with suggestions to use SSSG as a main component of soccer‐based programs for health promotion, as it induces high‐intensity exercise [18, 20, 24]. In this study, different strategies were used to promote time at high intensity and to organize the training session according to HIIT principles [44], which have been proposed to be more effective for BMI [45], insulin resistance [7, 45], SBP [14, 45], and CRF improvement [14, 45] in children and adolescents with obesity. Although the benefits of HIIT are commonly attributed to high intensity [46], some studies implementing isocaloric exercise protocols have found no differences between high‐ and low‐intensity exercise programs on measures of abdominal obesity or glucose metabolism [47, 48]. In this context, the superior cardiometabolic effects observed in SSSG compared with TSG may reflect greater accumulated training stimulus. While energy expenditure was not directly measured, increasing intensity or volume within the TSG format might have resulted in adaptations comparable to those achieved with SSSG.

Previous systematic reviews have suggested that recreational soccer programs effectively improve physical fitness in children and adolescents [18, 20]. However, our results showed no significant changes in CRF or muscle fitness in any of the exercise groups at the end of the intervention. Nevertheless, both exercise groups showed percentage improvements over time in the 1‐mile run/walk test (7% and 8%) and in horizontal jump performance (6% and 4%), although these changes did not reach statistical significance. Cvetkovic et al. [49], Seabra et al. [21], and Vasconcellos et al. [22] reported significant improvements in CRF after a recreational soccer‐based intervention for children with obesity. However, only Vasconcellos et al. [22] reported significant changes compared with the CG. In addition, only Cvetkovic et al. [49] included measures of lower limb strength and found no significant differences after the intervention. These heterogeneous results have also been described in previous systematic reviews assessing the effectiveness of exercise on measures of physical fitness in adolescents [45, 50] and could result from the physical and hormonal changes associated with puberty.

In addition, the low attendance found in our study could have influenced the results of physical fitness and other measures of cardiometabolic risk. The average attendance was less than 50% in both groups, and only 8 participants attended more than 50% of the planned sessions. Adherence and dropouts to training programs have not always been adequately reported in the literature [15, 18], and similar studies have mainly used a per‐protocol approach and did not report attendance [18]. Some studies have used an attendance of over 50% of the sessions as inclusion criteria for the final analysis [49, 51], which could be associated with lower attendance during the programs and, consequently, could lead to bias in the interpretation of the intervention efficacy and effectiveness. Although it has been proposed that SSSG can promote adherence because of its popularity [19, 20], the context in which it is implemented or from which participants are recruited can significantly influence adherence. For example, in contrast to our study, Seabra et al. [21] recruited children from an ambulatory obesity center and reported attendance of over 85% and only one dropout from a soccer‐based program. It is possible that the characteristics of the participants in this study favored greater commitment and motivation to adhere to the intervention.

During adolescence, the effectiveness of obesity management programs is often low [3, 4]. Adolescents tend to make impulsive decisions to seek immediate rewards rather than long‐term benefits [52]. This behavior reduces their receptivity to adult advice and affects their participation in health interventions such as our exercise program. Additionally, it is common for physical activity levels to decrease during this life stage [53], further complicating obesity management, especially in programs that include physical exercise components. Low attendance in our study may also have been affected by the residual consequences of the COVID‐19 pandemic on healthy behaviors such as daily physical activity [54]. For example, in the region where the intervention was implemented, 39% of male schoolchildren exhibited severe school absenteeism (less than 85% attendance) [55]. Although we did not collect objective data on school absenteeism, we believe that adolescents’ behavioral changes and COVID‐19 residual effects might have influenced attendance at our program, which was conducted immediately after school hours. Furthermore, the intervention was conducted during the autumn–winter season; although sessions took place indoors, seasonal factors such as reduced daylight and general activity patterns may have indirectly influenced attendance.

The results of this study suggest that SSSG may offer meaningful benefits for improving adolescents’ health. By directly comparing two soccer‐based training formats within a population with increased metabolic risk, our findings provide practical insight into how manipulating training structure may influence cardiometabolic adaptations. Therefore, high‐intensity SSSG may represent a useful, scalable, and low‐cost strategy that could be implemented in schools and recreational sports programs to promote exercise participation in this vulnerable population.

The results of this study, however, should be interpreted acknowledging some limitations of our study design: (i) This study did not control for confounding variables such as caloric intake or participants’ daily physical activity, which could have affected the results obtained; (ii) participants attended classes in different schools, so they were engaged in distinct physical education classes with different types of activities; (iii) energy expenditure in each exercise intervention was not assessed; therefore, there is no guarantee that programs were isocaloric, which limits the interpretation of exercise‐associated benefits; (iv) a limitation of the study is that additional measures of exercise intensity (e.g., RPE or distance covered at different speed zones) were not recorded; therefore, exercise intensity was characterized only by heart rate responses; and (v) the intervention was performed after school hours, which may have contributed to the low attendance.

## 5. Conclusion

After a 16‐week intervention in adolescents at metabolic risk, high‐intensity SSSG were associated with more beneficial effects on overall cardiometabolic health, body composition, and lipid profile than traditional soccer training. Compared with the CG, both exercise formats improved fat‐free mass, whereas only SSSG reduced fat mass percentage. By directly contrasting two soccer‐based training structures in a high‐risk adolescent population, these findings suggest that manipulating training design within soccer may enhance cardiometabolic adaptations. However, adherence to the exercise intervention was low in both groups, highlighting the need for future research to develop strategies that improve participation and maximize intervention effectiveness.

## Funding

This study has been supported by the Research Department, Universidad Adventista de Chile (Regular Research Project No. 2023‐183), and the National Fund for the Promotion of Sport (FONDEPORTE) of the Chilean Ministry of Sport (No. 2300120030). Nicolás Gómez‐Álvarez is supported by the Chilean National Agency for Research and Development (Agencia Nacional de Investigación y Desarrollo [ANID]) of Chile‐2019 (grant number 72200210). The Research Center in Physical Activity, Health and Leisure (CIAFEL), Faculty of Sport, University of Porto (FADEUP), and the Laboratory for Integrative and Translational Research in Population Health (ITR) are funded by the Portuguese Fundação para a Ciência e Tecnologia (FCT) Grants UIDB/00617/2020: doi:10.54499/UIDB/00617/2020, UIDP/00617/2020: doi: 10.54499/UIDP/00617/2020 and LA/P/0064/2020.

## Disclosure

This work has been previously published in the doctoral thesis of the first author [56].

## Conflicts of Interest

The authors declare no conflicts of interest.

## Supporting Information

Additional supporting information can be found online in the Supporting Information section.

## Supporting information


**Supporting Information** Supporting table 1. Time effects of SSSG and TSG on anthropometry, body composition, cardiometabolic risk factors, and health‐related physical fitness; Supporting table 2. Intervention effects of SSSG and TSG on anthropometry, body composition, cardiometabolic risk factors, and health‐related physical fitness; Supporting table 3. Results by attendance at exercise sessions for anthropometry and body composition; Supporting table 4. Results by attendance at exercise sessions for health‐related physical fitness; Supporting table 5. Results by attendance at exercise sessions for cardiometabolic risk factors.

## Data Availability

The data that support the findings of this study are available from the corresponding author upon reasonable request.
