# Supplementary material for: Effectiveness of High‐Intensity Small‐Sided Soccer Games Versus Traditional Soccer Training on Cardiovascular and Metabolic Health Outcomes in Adolescents With Increased Metabolic Risk: A Randomized Controlled Trial
Source: Transl Sports Med. 2026 May 13;2026:1078783. doi: 10.1155/tsm2/1078783 (PMC13169501; doi:10.1155/tsm2/1078783)
Supplement: Supplementary file 1 — Supporting Information Supporting table 1. Time effects of SSSG and TSG on anthropometry, body composition, cardiometabolic risk factors, and health‐related physical fitness; Supporting table 2. Intervention effects of SSSG and TSG on anthropometry, body composition, cardiometabolic risk factors, and health‐related physical fitness; Supporting table 3. Results by attendance at exercise sessions for anthropometry and body composition; Supporting table 4. Results by attendance at exercise sessions for health‐related physical fitness; Supporting table 5. Results by attendance at exercise sessions for cardiometabolic risk factors. [file TSM2-2026-1078783-s001.zip › RCT-consort.pdf]

# CONSORT 2010 checklist of information to include when reporting a randomised trial*

| Section/Topic | Item No | Checklist item | Reported on page No |
|---|---|---|---|
| **Title and abstract** | | | |
| | 1a | Identification as a randomised trial in the title | Page 1, line 3 |
| | 1b | Structured summary of trial design, methods, results, and conclusions (for specific guidance see CONSORT for abstracts) | Page 2, line 32 |
| **Introduction** | | | |
| Background and objectives | 2a | Scientific background and explanation of rationale | Page 3, line 63 |
| | 2b | Specific objectives or hypotheses | Page 4, line 97 |
| **Methods** | | | |
| Trial design | 3a | Description of trial design (such as parallel, factorial) including allocation ratio | Page 4, line 104 |
| | 3b | Important changes to methods after trial commencement (such as eligibility criteria), with reasons | |
| Participants | 4a | Eligibility criteria for participants | Page 4, line 114 |
| | 4b | Settings and locations where the data were collected | Page 4, line 112 |
| Interventions | 5 | The interventions for each group with sufficient details to allow replication, including how and when they were actually administered | Page 5, line 136 |
| Outcomes | 6a | Completely defined pre-specified primary and secondary outcome measures, including how and when they were assessed | Page 6, line 170 |
| | 6b | Any changes to trial outcomes after the trial commenced, with reasons | |
| Sample size | 7a | How sample size was determined | Page 8, line 233 |
| | 7b | When applicable, explanation of any interim analyses and stopping guidelines | |
| Randomisation: | | | |
| Sequence generation | 8a | Method used to generate the random allocation sequence | Page 5, line 130 |
| | 8b | Type of randomisation; details of any restriction (such as blocking and block size) | Page 5, line 130 |
| Allocation concealment mechanism | 9 | Mechanism used to implement the random allocation sequence (such as sequentially numbered containers), describing any steps taken to conceal the sequence until interventions were assigned | Page 5, line 130 |
| Implementation | 10 | Who generated the random allocation sequence, who enrolled participants, and who assigned participants to interventions | |
| Blinding | 11a | If done, who was blinded after assignment to interventions (for example, participants, care providers, those | |

| | | | |
|---|---|---|---|
| | | assessing outcomes) and how | |
| | 11b | If relevant, description of the similarity of interventions | |
| Statistical methods | 12a | Statistical methods used to compare groups for primary and secondary outcomes | Page 8, line 237 |
| | 12b | Methods for additional analyses, such as subgroup analyses and adjusted analyses | Page 8, line 237 |
| **Results** | | | |
| Participant flow (a diagram is strongly recommended) | 13a | For each group, the numbers of participants who were randomly assigned, received intended treatment, and were analysed for the primary outcome | Page 9, line 266 |
| | 13b | For each group, losses and exclusions after randomisation, together with reasons | Page 10, line 282 |
| Recruitment | 14a | Dates defining the periods of recruitment and follow-up | |
| | 14b | Why the trial ended or was stopped | |
| Baseline data | 15 | A table showing baseline demographic and clinical characteristics for each group | Page 10, line 294 |
| Numbers analysed | 16 | For each group, number of participants (denominator) included in each analysis and whether the analysis was by original assigned groups | Page 9, line 280 |
| Outcomes and estimation | 17a | For each primary and secondary outcome, results for each group, and the estimated effect size and its precision (such as 95% confidence interval) | Page 10, line 294 |
| | 17b | For binary outcomes, presentation of both absolute and relative effect sizes is recommended | Page 12, line 347 |
| Ancillary analyses | 18 | Results of any other analyses performed, including subgroup analyses and adjusted analyses, distinguishing pre-specified from exploratory | |
| Harms | 19 | All important harms or unintended effects in each group (for specific guidance see CONSORT for harms) | |
| **Discussion** | | | |
| Limitations | 20 | Trial limitations, addressing sources of potential bias, imprecision, and, if relevant, multiplicity of analyses | Page 16, line 493 |
| Generalisability | 21 | Generalisability (external validity, applicability) of the trial findings | Page 16, line 489 |
| Interpretation | 22 | Interpretation consistent with results, balancing benefits and harms, and considering other relevant evidence. | Page 13, line 391 |
| **Other information** | | | |
| Registration | 23 | Registration number and name of trial registry | Page 4, line 108 |
| Protocol | 24 | Where the full trial protocol can be accessed, if available | |
| Funding | 25 | Sources of funding and other support (such as supply of drugs), role of funders | Page 17, line 521 |

Citation: Schulz KF, Altman DG, Moher D, for the CONSORT Group. CONSORT 2010 Statement: updated guidelines for reporting parallel group randomised trials. BMC Medicine. 2010;8:18.
© 2010 Schulz et al. This is an Open Access article distributed under the terms of the Creative Commons Attribution License (http://creativecommons.org/licenses/by/2.0), which permits unrestricted use, distribution, and reproduction in any medium, provided the original work is properly cited.

*We strongly recommend reading this statement in conjunction with the CONSORT 2010 Explanation and Elaboration for important clarifications on all the items. If relevant, we also recommend reading CONSORT extensions for cluster randomised trials, non-inferiority and equivalence trials, non-pharmacological treatments, herbal interventions, and pragmatic trials. Additional extensions are forthcoming: for those and for up-to-date references relevant to this checklist, see www.consort-statement.org.

# CONSORT Harms 2022 integrated into CONSORT 2010 items checklist of information to include when reporting a randomised trial

| Section/Topic | Item No | Checklist item | Reported on page No |
|---|---|---|---|
| **Title and abstract** | | | |
| | 1a | Identification as a randomised trial in the title | Page 1, line 3 |
| | 1b | Structured summary of trial design, methods, results of outcomes of benefits and harms, and conclusions (for specific guidance see CONSORT for abstracts) | Page 2, line 32 |
| **Introduction** | | | |
| Background and objectives | 2a | Scientific background and explanation of rationale | Page 3, line 63 |
| | 2b | Specific objectives or hypotheses for outcomes benefits and harms | Page 4, line 97 |
| **Methods** | | | |
| Trial design | 3a | Description of trial design (such as parallel, factorial) including allocation ratio | Page 4, line 104 |
| | 3b | Important changes to methods after trial commencement (such as eligibility criteria), with reasons | |
| Participants | 4a | Eligibility criteria for participants | Page 4, line 114 |
| | 4b | Settings and locations where the data were collected | Page 4, line 112 |
| Interventions | 5 | The interventions for each group with sufficient details to allow replication, including how and when they were actually administered | Page 5, line 136 |
| Outcomes | 6a | Completely defined pre-specified primary and secondary outcome measures for both benefits and harms, including how and when they were assessed | Page 6, line 170 |
| | 6b | Any changes to trial outcomes after the trial commenced, with reasons | |
| | 6c | Describe if and how non-prespecified outcomes of benefits and harms were identified, including any selection criteria, if applicable | |
| Sample size | 7a | How sample size was determined | Page 8, line 233 |
| | 7b | When applicable, explanation of any interim analyses and stopping guidelines | |

| Section/Topic | Item No | Checklist item | Reported on page No |
|---|---|---|---|
| Randomisation: | | | |
| Sequence generation | 8a | Method used to generate the random allocation sequence | Page 4, line 100 |
| | 8b | Type of randomisation; details of any restriction (such as blocking and block size) | Page 4, line 100 |
| Allocation concealment mechanism | 9 | Mechanism used to implement the random allocation sequence (such as sequentially numbered containers), describing any steps taken to conceal the sequence until interventions were assigned | Page 4, line 104 |
| Implementation | 10 | Who generated the random allocation sequence, who enrolled participants, and who assigned participants to interventions | |
| Blinding | 11a | If done, who was blinded after assignment to interventions (e.g., participants, care providers, those assessing outcomes of benefits and harms) and how | |
| | 11b | If relevant, description of the similarity of interventions | |
| Statistical methods | 12a | Statistical methods used to compare groups for primary and secondary outcomes of both benefits and harms | Page 8, line 237 |
| | 12b | Methods for additional analyses, such as subgroup analyses and adjusted analyses | |
| **Results** | | | |
| Participant flow (a diagram is strongly recommended) | 13a | For each group, the numbers of participants who were randomly assigned, received intended treatment, and were analysed for outcomes of benefits and harms | Page 9, line 280 |
| | 13b | For each group, losses and exclusions after randomisation, together with reasons | Page 9, line 280 |
| Recruitment | 14a | Dates defining the periods of recruitment and follow-up for outcomes of benefits and harms | |
| | 14b | Why the trial ended or was stopped | |
| Baseline data | 15 | A table showing baseline demographic and clinical characteristics for each group | Page 10, line 294 |
| Numbers analysed | 16 | For each group, number of participants (denominator) included in each analysis and whether the analysis was by original assigned groups and if any exclusions were made | Page 9, line 280 |
| Outcomes and estimation | 17a | For each primary and secondary outcome of benefits and harms, results for each group, and the estimated effect size and its precision (such as 95% confidence interval) | Page 9, line 296 |
| | 17a2 | For outcomes omitted from the trial report (benefits and harms), provide rationale for not reporting and indicate where the data on omitted outcomes can be accessed | |
| | 17b | Presentation of both absolute and relative effect sizes is recommended, for outcomes of benefits and harms | Page 11, line 335 |
| | 17c | Report zero events if no harms were observed | |
| Ancillary analyses | 18 | Results of any other analyses performed, including subgroup analyses and adjusted analyses, distinguishing pre-specified from exploratory | Page 11, line 358 |
| Harms | 19 | All important harms or unintended effects in each group (for specific guidance see CONSORT for harms) | |

| Section/Topic | Item No | Checklist item | Reported on page No |
|---|---|---|---|
| **Discussion** | | | |
| Limitations | 20 | Trial limitations, addressing sources of potential bias related to the approach to collecting or reporting data on harms, imprecision, and, if relevant, multiplicity or selection of analyses | Page 16, line 493 |
| Generalisability | 21 | Generalisability (external validity, applicability) of the trial findings | Page 16, line 489 |
| Interpretation | 22 | Interpretation consistent with results, balancing benefits and harms, and considering other relevant evidence | Page 12, line 391 |
| **Other information** | | | |
| Registration | 23 | Registration number and name of trial registry | Page 4, line 108 |
| Protocol | 24 | Where the full trial protocol and other relevant documents can be accessed, including additional data on harms | |
| Funding | 25 | Sources of funding and other support (such as supply of drugs), role of funders | Page 17, line 521 |

Note: Adapted from Schulz (2010) to integrate items of CONSORT Harms 2022 (Junqueira 2022) [https://creativecommons.org/licenses/by/2.0/]. CONSORT items 1b, 2b, 6a, 11a, 12a, 13a, 14a, 16a, 17a, 17b, 18, 20 and 24 of were modified to incorporate elements relevant to the reporting of harms. Two new items were added (item 6c and 17a2). Please see the CONSORT Harms 2022 statement for additional details (Junqueira 2022).

We strongly recommend reading the CONSORT 2010 statement (Schulz 2010) in conjunction with the CONSORT Harms 2022 statement (Junqueira 2022) for important clarifications on all the items. If relevant, we also recommend reading CONSORT extensions for cluster randomised trials, non-inferiority and equivalence trials, non-pharmacological treatments, adaptive designs, pilot and feasibility studies, multi arm trials, cross-over and pragmatic trials. Additional extensions are forthcoming: for those and for up-to-date references relevant to this checklist, see EQUATOR Network.

**References**

Junqueira DR, Zorzela L, Golder S, Loke Y, Gagnier JJ, Julious SA, Li T, Mayo-Wilson E, Pham B, Phillips R, Santaguida P, Scherer RW, Gøtzsche PC, Moher D, Ioannidis JPA and Vohra S on behalf of the CONSORT Harms Group. CONSORT Harms 2022 statement, explanation, and elaboration: updated guideline for the reporting of harms in randomised trials. *BMJ* 2023 **381:** e073725 DOI 10.1136/bmj-2022-073725

Schulz KF, Altman DG, Moher D, for the CONSORT Group. CONSORT 2010 Statement: updated guidelines for reporting parallel group randomised trials. *BMJ* 2010 **340:**c332 doi: 10.1136/bmj.c332.
