# Supplementary material for: Effectiveness of High‐Intensity Small‐Sided Soccer Games Versus Traditional Soccer Training on Cardiovascular and Metabolic Health Outcomes in Adolescents With Increased Metabolic Risk: A Randomized Controlled Trial
Source: Transl Sports Med. 2026 May 13;2026:1078783. doi: 10.1155/tsm2/1078783 (PMC13169501; doi:10.1155/tsm2/1078783)
Supplement: Supplementary file 1 — Supporting Information Supporting table 1. Time effects of SSSG and TSG on anthropometry, body composition, cardiometabolic risk factors, and health‐related physical fitness; Supporting table 2. Intervention effects of SSSG and TSG on anthropometry, body composition, cardiometabolic risk factors, and health‐related physical fitness; Supporting table 3. Results by attendance at exercise sessions for anthropometry and body composition; Supporting table 4. Results by attendance at exercise sessions for health‐related physical fitness; Supporting table 5. Results by attendance at exercise sessions for cardiometabolic risk factors. [file TSM2-2026-1078783-s001.zip › Supplementary Material.pdf]

Effectiveness of high-intensity small-sided soccer games versus traditional soccer training on cardiovascular and metabolic health outcomes in adolescents with increased metabolic risk - a randomized controlled trial

Nicolás Gómez-Álvarez[1-2-*]; Felipe Hermosilla-Palma[3]; Tomás Reyes-Amigo[4]; Mauricio Díaz-Alvarado[5]; Cristian Luarte-Rocha[6]; Juan Pablo Espinoza-Puelles[7]; Rafael Zapata-Lamana[8-9]; Carlos Cristi-Montero[7]; José Oliveira[10-11]; Hélder Fonseca[10-11]

[1] Centre for Research, Education, Innovation and Intervention in Sport, Faculty of Sport of the University of Porto, 4200-450 Porto, Portugal

[2] Centro Regional de Estudios Avanzados en Estilos de Vida Activos y Saludables, Universidad Adventista de Chile, Chillán, Chile; nicolasgomez@unach.cl

[3] Pedagogía en Educación Física, Facultad de Educación, Universidad Autónoma de Chile, Talca, Chile: felipe.hermosilla@uautonoma.cl

[4] Physical Activity Sciences Observatory (OCAF), Department of Physical Activity Sciences, Universidad de Playa Ancha, Valparaíso, Chile; tomas.reyes@upla.cl

[5] Departamento de Educación y Humanidades, Universidad de Magallanes, Punta Arenas, Chile; mauricio.diaz@umag.cl

[6] Facultad de Odontología y Ciencias de la Rehabilitación, Universidad de San Sebastían, Concepción 4080871, Chile; cristian.luarte@uss.cl

[7] IRyS Group, Physical Education School, Pontificia Universidad Católica de Valparaíso, Valparaíso, Chile; carlos.cristi.montero@gmail.com (C.C-M), espinoza.puelles.jp@gmail.com (J.P.E-P)

[8] Escuela de Kinesiología, Facultad de Salud, Universidad Santo Tomás, Los Ángeles 4441171, Chile; rzapatal@santotomas.cl

[9] Escuela de Educación, Universidad de Concepción, Los Ángeles 4451032, Chile

[10] Research Centre in Physical Activity, Health, and Leisure (CIAFEL), Faculty of Sport, University of Porto, 4200-450 Porto, Portugal; hfonseca@fade.up.pt (H.F); joliveira@fade.up.pt (J.O.)

[11] Laboratory for Integrative and Translational Research in Population Health (ITR), Porto, Portugal

* Correspondence: nicolasgomez@unach.cl

Supplementary table 1. Time effects of SSSG and TSG on anthropometry, body composition, cardiometabolic risk factors and health-related physical fitness

	**Time effects (pre-post)**		
	**CG<br>Pre/post ratio (IC95%); p-value**	**SSSG<br>pre/post ratio (IC95%); p-value**	**TSG<br>Pre/post ratio (IC95%); p-value**
Anthropometry and body composition			
Weight (kg)	0.99 (0.97 to 1.03); 1.00	1.01 (0.973 to 1.04); 1.00	0.99 (0.96 to 1.03); 1.00
Height (cm)	**0.98 (0.97 to 0.99); <0.0001**	**0.99 (0.98 to 0.99); <.0001**	**0.97 (0.96 to 0.99); <.0001**
BMI (kg/m2)	1.02 (0.99 to 1.06); 0.28	**1.03 (1.00 to 1.06); 0.02**	1.01 (0.98 to 1.04); 1.00
BMI Z-score	**1.06 (1.01 to 1.12); 0.02**	**1.08 (1.02 to 1.14); <.001**	1.05 (0.99 to 1.10); 0.30
Waist circumference (cm)	1.01 (0.97 to 1.04); 1.00	1.03 (0.99 to 1.06); 0.16	1.01 (0.97 to 1.04); 1.00
WtHR	1.02 (0.98 to 1.06); 1.00	1.04 (0.99 to 1.08); 0.06	1.03 (0.99 to 1.07); 0.62
Fat mass (kg)	0.95 (0.87 to 1.03); 1.00	1.04 (0.95 to 1.13); 1.00	0.98 (0.90 to 1.07); 1.00
Fat mass (%)	0.98 (0.95 to 1.02); 1.00	1.03 (0.99 to 1.07); 0.24	0.99 (0.96 to 1.04); 1.00
Fat-free mass (kg)	1.02 (1.00 to 1.05); 0.05	0.99 (0.97 to 1.01); 1.00	**0.97 (0.95 to 0.99); 0.002**
Muscle mass (kg)	0.99 (0.97 to 1.03); 1.00	0.99 (0.96 to 1.02); 1.00	0.98 (0.95 to 1.01); 0.89
Cardiometabolic risk factors			
Heart rate (bpm)	1.02 (0.94 to 1.10); 1.00	**1.08 (1.01 to 1.17); 0.04**	1.05 (0.97 to 1.14); 0.99
Systolic blood pressure (mmHg)	1.05 (0.99 to 1.11); 0.06	1.04 (0.98 to 1.09); 0.69	1.00 (0.95 to 1.06); 1.00
diastolic blood pressure (mmHg)	1.04 (0.96 to 1.11); 1.00	1.06 (0.99 to 1.13); 0.09	1.05 (0.98 to 1.12); 0.74
Insuline	1.08 (0.78 to 1.49); 1.00	1.23 (0.89 to 1.71); 0.90	0.82 (0.60 to 1.13); 1.00
HOMA-IR	1.05 (0.74 to 1.49); 1.00	1.22 (0.85 to 1.75); 1.00	0.79 (0.56 to 1.12); 0.75
HbA1c (%)	0.99 (0.97 to 1.01); 1.00	0.98 (0.97 to 1.00); 0.06	**0.96 (0.95 to 0.98); <.0001**
Glucose (mg/dl)	0.98 (0.93 to 1.02); 1.00	1.00 (0.96 to 1.05); 1.00	0.96 (0.92 to 1.00); 0.06
Total cholesterol (mg/dl)	**1.13 (1.06 to 1.20); <.0001**	**1.13 (1.07 to 1.21); <.0001**	1.06 (0.99 to 1.12); 0.15
HDL-cholesterol (mg/dl)	1.07 (0.99 to 1.15); 0.14	1.04 (0.97 to 1.12); 1.00	0.99 (0.92 to 1.06); 1.00
LDL-cholesterol (mg/dl)	**1.14 (1.03 to 1.26); <.01**	**1.15 (1.043 to 1.27); <.001**	1.09 (0.98 to 1.20); 0.14
VLDL-cholesterol (mg/dl)	1.24 (0.99 to 1.55); 0.06	**1.35 (1.09 to 1.67); <.001**	1.03 (0.83 to 1.27); 1.00
Triglyceride (mg/dl)	1.23 (0.99 to 1.53); 0.09	**1.35 (1.09 to 1.67); <.001**	1.03 (0.83 to 1.28); 1.00
AST (U/L)	0.91 (0.79 to 1.04); 0.57	0.89 (0.78 to 1.01); 0.12	**0.86 (0.76 to 0.99); 0.02**
ALT (U/L)	0.84 (0.65 to 1.09); 0.82	0.84 (0.65 to 1.09); 0.69	0.89 (0.69 to 1.16); 1.00
AST/ALT ratio	1.07 (0.90 to 1.27); 1.00	1.04 (0.88 to 1.23); 1.00	0.93 (0.79 to 1.10); 1.00
GGT (U/L)	**1.29 (1.06 to 1.58); <.01**	**1.28 (1.05 to 1.55); <.001**	**1.24 (1.02 to 1.51); 0.02**
SPISE	0.94 (0.87 to 1.01); 0.10	**0.89 (0.83 to 0.96); <.0001**	0.98 (0.91 to 1.05); 1.00
Health-related physical fitness			
1-mile run/walk test (min)	1.01 (0.93 to 1.10); 1.00	1.07 (0.99 to 1.15); 0.23	1.08 (0.99 to 1.17); 0.08
Handgrip strength (kg)	0.99 (0.94 to 1.03); 1.00	0.97 (0.93 to 1.01); 0.27	0.96 (0.92 to 1.01); 0.34
Horizontal jump (cm)	0.99 (0.93 to 1.07); 1.00	0.95 (0.88 to 1.01); 0.24	0.98 (0.91 to 1.05); 1.00

CG, Control group; SSSG, small-sided soccer games group; TSG, Traditional soccer group; BMI, body mass index; WC, WtHR, waist to height ratio; HOMA-IR, homeostatic model assessment for insulin resistance; HbA1c, glycated hemoglobin A1c, TC, total cholesterol; HDL-C, high-density lipoprotein cholesterol; LDL-C, low-density lipoprotein cholesterol; VLDL-C, very low-density lipoprotein cholesterol; TG, triglycerides; ALT, alanine aminotransferase; AST, aspartate aminotransferase; GGT, γ-glutamyl transpeptidase; SPISE, single-point insulin sensitivity estimator.

Note: Results for the effect of time (difference pre-post intervention) are presented as ratio of the estimated means with a 95% confidence interval. Models adjusted for baseline values of the dependent variable and peak height velocity.
Ratios <1 indicate a reduction from baseline to post-intervention, whereas ratios >1 indicate an increase.

Supplementary table 2. Intervention effects of SSSG and TSG on anthropometry, body composition, cardiometabolic risk factors and health-related physical fitness

	Intervention effects		
	**CG / SSSG ratio (IC95%); p-value**	**CG / TSG ratio (IC95%); p-value**	**SSSG / TSG ratio (IC95%); p-value**
Anthropometry and body composition			
Weight	0.99 (0.96 to 1.03); 1.00	1.00 (0.97 to 1.04); 1.00	1.01 (0.97 to 1.05); 1.00
Height	1.00 (0.99 to 1.01); 0.26	1.00 (0.99 to 1.01); 0.36	0.99 (0.99 to 1.00); 1.00
BMI	0.99 (0.96 to 1.03); 1.00	1.02 (0.98 to 1.05); 0.73	1.03 (0.99 to 1.06); 0.23
BMI Z-score	1.00 (0.99 to 1.01); 0.26	1.00 (0.99 to 1.01); 0.36	0.99 (0.99 to 1.00); 1.00
Waist circumference	0.98 (0.94 to 1.02); 0.45	1.00 (0.96 to 1.04); 1.00	1.02 (0.99 to 1.06); 0.43
WtHR	0.98 (0.93 to 1.02); 0.77	0.99 (0.94 to 1.04); 1.00	1.01 (0.97 to 1.06); 1.00
Fat mass	0.91 (0.83 to 1.01); 0.09	0.97 (0.88 to 1.07); 1.00	1.06 (0.96 to 1.17); 0.43
Fat mass	**0.96 (0.91 to 0.99); 0.03**	0.99 (0.95 to 1.03); 1.00	1.03 (0.99 to 1.08); 0.20
Fat-free mass	**1.04 (1.01 to 1.06); <.01**	**1.05 (1.03 to 1.08); <.0001**	1.02 (0.99 to 1.05); 0.29
Muscle mass	1.01 (0.97 to 1.04); 1.00	1.02 (0.98 to 1.06); 0.77	1.01 (0.97 to 1.05); 1.00
Cardiometabolic risk factors			
Heart rate	0.94 (0.86 to 1.03); 0.33	0.97 (0.89 to 1.06); 1.00	1.03 (0.94 to 1.13); 1.00
Systolic blood pressure	1.02 (0.96 to 1.08); 1.00	1.05 (0.99 to 1.11); 0.19	1.03 (0.97 to 1.09); 0.65
diastolic blood pressure	0.98 (0.91 to 1.06); 1.00	0.99 (0.91 to 1.08); 1.00	1.01 (0.94 to 1.09); 1.00
Insuline	0.88 (0.60 to 1.27); 1.00	1.33 (0.91 to 1.91); 0.22	**1.50 (1.04 to 2.18); 0.03**
HOMA-IR	0.86 (0.57 to 1.29); 1.00	1.33 (0.89 to 1.99); 0.27	**1.55 (1.03 to 2.32); 0.03**
HbA1c	1.01 (0.99 to 1.03); 1.00	**1.03 (1.01 to 1.05); 0.01**	1.02 (0.99 to 1.04); 0.08
Glucose	0.97 (0.92 to 1.02); 0.55	1.02 (0.97 to 1.07); 1.00	1.05 (0.99 to 1.10); 0.08
Total cholesterol	0.99 (0.93 to 1.07); 1.00	1.07 (0.99 to 1.15); 0.08	**1.07 (1.00 to 1.15); 0.04**
HDL-cholesterol	1.03 (0.94 to 1.12); 1.00	1.08 (0.99 to 1.17); 0.10	1.05 (0.97 to 1.14); 0.48
LDL-cholesterol	0.99 (0.88 to 1.11); 1.00	1.05 (0.93 to 1.17); 1.00	1.06 (0.94 to 1.18); 0.79
VLDL-cholesterol	0.92 (0.71 to 1.18); 1.00	1.21 (0.90 to 1.55); 0.21	**1.31 (1.02 to 1.68); 0.02**
Triglyceride	0.91 (0.71 to 1.17); 1.00	1.20 (0.93 to 1.54); 0.27	**1.31 (1.02 to 1.67); 0.03**
SPISE	1.05 (0.96 to 1.14); 0.61	0.95 (0.88 to 1.04); 0.49	**0.91 (0.84 to 0.99); 0.02**
GGT	1.01 (0.81 to 1.27); 1.00	1.04 (0.83 to 1.30); 1.00	1.02 (0.82 to 1.28); 1.00
AST/ALT ratio	1.02 (0.84 to 1.25); 1.00	1.15 (0.94 to 1.39); 0.28	1.12 (0.92 to 1.36); 0.49
Health-related physical fitness			
1-mile run/walk test	0.95 (0.86 to 1.04); 0.57	0.94 (0.85 to 1.03); 0.34	0.99 (0.90 to 1.08); 1.00
Handgrip strength	1.02 (0.97 to 1.07); 0.99	1.02 (0.97 to 1.07); 1.00	1.00 (0.95 to 1.05); 1.00
Horizontal jump	1.06 (0.97 to 1.14); 0.35	1.02 (0.94 to 1.11); 1.00	0.97 (0.90 to 1.05); 1.00

CG, Control group; SSSG, small-sided soccer games group; TSG, Traditional soccer group; BMI, body mass index; WC, WtHR, waist to height ratio; HOMA-IR, homeostatic model assessment for insulin resistance; HbA1c, glycated hemoglobin A1c, TC, total cholesterol; HDL-C, high-density lipoprotein cholesterol; LDL-C, low-density lipoprotein cholesterol; VLDL-C, very low-density lipoprotein cholesterol; TG, triglycerides; ALT, alanine aminotransferase; AST, aspartate aminotransferase; GGT, γ-glutamyl transpeptidase; SPISE, single-point insulin sensitivity estimator.

Note: Results for the effect of time (difference between groups) are presented as ratio of the estimated means with a 95% confidence interval. Models adjusted for baseline values of the dependent variable and peak height velocity.
Ratios <1 indicate a reduction from baseline to post-intervention, whereas ratios >1 indicate an increase.

Supplementary table 3. Results by attendance at exercise sessions for Anthropometry and body composition

	Group	Baseline	Post-intervention	pre/post ratio (IC95%)	p-value	p-value between-group
Weight (kg)	CG	75.2 (73.5 to 76.9)	75.3 (73.5 to 77.2)	0.99 (0.96 to 1.04)	1.00	CG/SSSG-h = 1.00; CG/SSSG-l = 1.00; **CG/TSG-h = 0.008**; CG/TSG-l = 1.00; SSSG-h/SSSG-l = 1.00; TSG-h/TSG-l = 0.99; SSSG-h/TSG-h = 1.00; SSSG-h/TSG-l = 1.00;
	SSSG-h	75.4 (72.9 to 78.0)	74.6 (72.1 to 77.1)	1.01 (0.96 to 1.06)	1.00	
	SSSG-l	75.6 (73.3 to 78.0)	75.7 (73.1 to 78.3)	0.99 (0.95 to 1.05)	1.00	
	TSG-h	76.7 (74.1 to 79.4)	75.8 (73.2 to 78.4)	1.01 (0.96 to 1.06)	1.00	
	TSG-l	75.4 (73.0 to 77.9)	77.4 (74.5 to 80.3)	0.98 (0.92 to 1.03)	1.00	
Height (cm)	CG	162.7 (162.3 to 163.1)	164.5 (164.1 to 165.0)	0.99 (0.98 to 0.99)	**<.0001**	CG/SSSG-h = 1.00; CG/SSSG-l = 1.00; **CG/TSG-h = 0.008;** CG/TSG-l = 1.00; SSSG-h/SSSG-l = 1.00; **TSG-h/TSG-l = 0.002;** SSSG-h/TSG-h = 100; SSSG-h/TSG-l = 0.26;
	SSSG-h	162.5 (161.9 to 163.0)	164.9 (164.3 to 165.4)	0.99 (0.98 to 0.99)	**<.0001**	
	SSSG-l	162.6 (162.0 to 163.1)	165.0 (164.4 to 165.6)	0.98 (0.98 to 0.99)	**<.0001**	
	TSG-h	162.7 (162.1 to 163.2)	165.6 (165.0 to 166.2)	0.98 (0.98 to 0.99)	**<.0001**	
	TSG-l	162.6 (162.0 to 163.1)	164.1 (163.4 to 164.7)	0.99 (0.98 to 0.99)	**<.0001**	
BMI (kg/m^2)	CG	28.5 (28.0 to 29.0)	27.8 (27.3 to 28.4)	1.02 (0.99 to 1.06)	0.78	CG/SSSG-h = 1.00; CG/SSSG-l = 1.00; CG/TSG-h = 1.00; CG/TSG-l = 1.00; SSSG-h/SSSG-l = 1.00; SSSG-h/TSG-h = 1.00; SSSG-h/TSG-l = 0.29; SSSG-l/TSG-h = 1.00; SSSG-l/TSG-l = 1.00; TSG-h/TSG-l = 1.00;
	SSSG-h	28.7 (27.9 to 29.4)	27.6 (26.9 to 28.3)	1.04 (0.99 to 1.09)	0.18	
	SSSG-l	28.6 (27.9 to 29.3)	27.9 (27.1 to 28.7)	1.02 (0.98 to 1.07)	1.00	
	TSG-h	28.4 (27.7 to 29.2)	28.0 (27.2 to 28.7)	1.02 (0.97 to 1.06)	1.00	
	TSG-l	28.3 (27.6 to 29.0)	28.4 (27.6 to 29.3)	0.99 (0.95 to 1.04)	1.00	
BMI Z-score	CG	2.57 (2.47 to 2.67)	2.42 (2.32 to 2.52)	1.06 (1.00 to 1.13)	**0.05**	CG/SSSG-h = 1.00; CG/SSSG-l = 1.00; CG/TSG-h = 1.00; CG/TSG-l = 1.00; SSSG-h/SSSG-l = 1.00; SSSG-h/TSG-h = 1.00; SSSG-h/TSG-l = 1.00; SSSG-l/TSG-h = 1.00; SSSG-l/TSG-l = 1.00; TSG-h/TSG-l = 1.00;
	SSSG-h	2.59 (2.45 to 2.75)	2.38 (2.25 to 2.53)	1.09 (1.00 to 1.18)	**0.04**	
	SSSG-l	2.59 (2.46 to 2.73)	2.43 (2.29 to 2.57)	1.07 (0.98 to 1.17)	0.53	
	TSG-h	2.52 (2.38 to 2.68)	2.38 (2.25 to 2.53)	1.06 (0.97 to 1.15)	1.00	
	TSG-l	2.52 (2.39 to 2.67)	2.45 (2.30 to 2.61)	1.03 (0.94 to 1.13)	1.00	
WC (cm)	CG	92.8 (91.0 to 94.7)	92.3 (90.3 to 94.3)	1.01 (0.97 to 1.04)	1.00	CG/SSSG-h = 0.44; CG/SSSG-l = 1.00; CG/TSG-h = 1.00; CG/TSG-l = 1.00; SSSG-h/SSSG-l = 1.00; SSSG-h/TSG-h = 1.00; SSSG-h/TSG-l = 0.46; SSSG-l/TSG-h = 1.00; SSSG-l/TSG-l = 1.00; TSG-h/TSG-l = 1.00;
	SSSG-h	93.1 (90.3 to 95.9)	89.1 (86.5 to 91.8)	1.04 (0.99 to 1.10)	0.20	
	SSSG-l	92.8 (90.4 to 95.4)	91.8 (89.1 to 94.6)	1.01 (0.96 to 1.06)	1.00	
	TSG-h	92.8 (90.1 to 95.5)	91.8 (89.1 to 94.5)	1.01 (0.96 to 1.06)	1.00	
	TSG-l	92.4 (89.9 to 95.0)	92.6 (89.6 to 95.7)	0.99 (0.95 to 1.05)	1.00	
WtHr	CG	0.571 (0.557 to 0.584)	0.561 (0.547 to 0.576)	1.02 (0.98 to 1.06)	1.00	CG/SSSG-h = 1.00; CG/SSSG-l = 1.00; CG/TSG-h = 1.00; CG/TSG-l = 1.00; SSSG-h/SSSG-l = 1.00; SSSG-h/TSG-h = 1.00; SSSG-h/TSG-l = 1.00; SSSG-l/TSG-h = 1.00; SSSG-l/TSG-l = 1.00; TSG-h/TSG-l = 1.00;
	SSSG-h	0.573 (0.553 to 0.594)	0.545 (0.526 to 0.565)	1.05 (0.99 to 1.12)	0.25	
	SSSG-l	0.572 (0.553 to 0.590)	0.557 (0.538 to 0.578)	1.03 (0.96 to 1.09)	1.00	
	TSG-h	0.573 (0.553 to 0.594)	0.548 (0.529 to 0.567)	1.05 (0.99 to 1.11)	0.61	
	TSG-l	0.568 (0.550 to 0.587)	0.565 (0.543 to 0.587)	1.01 (0.94 to 1.08)	1.00	
Fat mass (kg)	CG	28.3 (26.6 to 30.0)	29.8 (27.9 to 31.8)	0.95 (0.86 to 1.04)	1.00	CG/SSSG-h = 0.47; CG/SSSG-l = 1.00; CG/TSG-h = 1.00; CG/TSG-l = 1.00; SSSG-h/SSSG-l = 1.00; SSSG-h/TSG-h = 1.00; SSSG-h/TSG-l = 1.00; SSSG-l/TSG-h = 1.00; SSSG-l/TSG-l = 1.00; TSG-h/TSG-l = 1.00;
	SSSG-h	28.4 (25.9 to 31.1)	27.1 (24.8 to 29.7)	1.02 (0.82 to 1.26)	1.00	
	SSSG-l	28.7 (26.4 to 31.2)	28.0 (25.5 to 30.7)	1.03 (0.89 to 1.18)	1.00	
	TSG-h	26.8 (24.4 to 29.4)	27.1 (24.7 to 29.7)	0.99 (0.870 to 1.13)	1.00	
	TSG-l	27.5 (25.2 to 29.9)	28.7 (26.0 to 31.7)	0.96 (0.83 to 1.11)	1.00	
Fat mass (%)	CG	37.3 (36.1 to 38.5)	37.9 (36.6 to 39.2)	0.99 (0.94 to 1.03)	1.00	CG/SSSG-h = 0.23; CG/SSSG-l = 0.53; CG/TSG-h = 1.00; CG/TSG-l = 1.00; SSSG-h/SSSG-l = 1.00; SSSG-h/TSG-h = 1.00; SSSG-h/TSG-l = 0.51; SSSG-l/TSG-h = 1.00; SSSG-l/TSG-l = 0.90; TSG-h/TSG-l = 1.00;
	SSSG-h	37.3 (35.5 to 39.1)	36.1 (34.4 to 37.8)	1.03 (0.98 to 1.09)	1.00	
	SSSG-l	37.1 (35.5 to 38.8)	36.1 (34.5 to 37.9)	1.03 (0.97 to 1.09)	1.00	
	TSG-h	37.0 (35.2 to 38.8)	36.6 (34.9 to 38.4)	1.01 (0.95 to 1.07)	1.00	
	TSG-l	36.3 (34.7 to 37.9)	36.9 (35.1 to 38.8)	0.98 (0.92 to 1.05)	1.00	
Fat-free mass (kg)	CG	47.1 (45.9 to 48.4)	46.0 (44.8 to 47.3)	1.02 (0.99 to 1.05)	0.12	CG/SSSG-h = 0.23; **CG/SSSG-l = 0.03; CG/TSG-h = <.0001; CG/TSG-l = 0.04;** SSSG-h/SSSG-l = 1.00; SSSG-h/TSG-h = 0.25; SSSG-h/TSG-l = 1.00; SSSG-l/TSG-h = 1.00; SSSG-l/TSG-l = 1.00; TSG-h/TSG-l = 1.00;
	SSSG-h	46.6 (44.8 to 48.5)	46.9 (45.0 to 48.8)	0.99 (0.96 to 1.03)	1.00	
	SSSG-l	46.6 (44.9 to 48.3)	47.4 (45.6 to 49.2)	0.98 (0.95 to 1.02)	1.00	
	TSG-h	46.6 (44.8 to 48.6)	48.5 (46.6 to 50.5)	0.96 (0.93 to 0.99)	**0.008**	
	TSG-l	46.3 (44.6 to 48.1)	47.1 (45.3 to 49.0)	0.98 (0.95 to 1.02)	1.00	
Muscle mass (kg)	CG	26.0 (25.2 to 26.9)	26.1 (25.2 to 27.0)	0.99 (0.96 to 1.03)	1.00	CG/SSSG-h = 1.00; CG/SSSG-l = 1.00; CG/TSG-h = 1.00; CG/TSG-l = 1.00; SSSG-h/SSSG-l = 1.00; SSSG-h/TSG-h = 1.00; SSSG-h/TSG-l = 1.00; SSSG-l/TSG-h = 1.00; SGb/TSG-l = 1.00; TSG-h/TSG-l = 1.00;
	SSSG-h	25.7 (24.4 to 27.0)	25.8 (24.5 to 27.1)	0.99 (0.95 to 1.04)	1.00	
	SSSG-l	25.7 (24.5 to 26.9)	26.1 (24.9 to 27.4)	0.98 (0.94 to 1.03)	1.00	
	TSG-h	25.9 (24.6 to 27.3)	26.5 (25.2 to 27.9)	0.98 (0.93 to 1.03)	1.00	
	TSG-l	25.4 (24.3 to 26.7)	25.9 (24.6 to 27.3)	0.98 (0.93 to 1.04)	1.00	

CG, Control group; SSSG-h, small-sided soccer games group with high attendance (over 50% attendance); SSSG-l, small-sided soccer games group with attendance low attendance (below 50% attendance); TSG-h, Traditional soccer group with high attendance (over 50% attendance); TSG-l, Traditional soccer group with low attendance (below 50% attendance); BMI, body mass index; WC, waist circumference; WtHR, waist to height ratio

Data: Estimated mean (confidence interval 95%). Models adjusted for baseline values of the dependent variable and peak height velocity.

Note: Ratios represent baseline-to-post comparisons (Pre/Post) of estimated marginal means derived from the generalized linear mixed model with gamma distribution and log link. Estimates are back-transformed from the log scale. Ratios <1 indicate a reduction from baseline to post-intervention, whereas ratios >1 indicate an increase.

Supplementary table 4. Results by attendance at exercise sessions for h*ealth-related physical fitness*

	Group	Baseline	Post-intervention	pre/post ratio (IC95%)	p-value	p-value between-group
1-mile walk/run test (min)	CG	13.3 (12.7 to 14.0)	13.2 (12.5 to 13.9)	1.01 (0.92 to 1.11)	1.00	CG/SSSG-h = 1.00; CG/SSSG-l = 1.00; CG/TSG-h = 1.00; CG/TSG-l = 0.72; SSSG-h/SSSG-l = 1.00; SSSG-h/TSG-h = 1.00; SSSG-h/TSG-l = 1.00; SSSG-l/TSG-h = 1.00; SSSG-l/TSG-l = 1.00; TSG-h/TSG-l = 1.00;
	SSSG-h	13.3 (12.4 to 14.2)	12.6 (11.8 to 13.5)	1.05 (0.93 to 1.19)	1.00	
	SSSG-l	13.3 (12.5 to 14.1)	12.3 (11.4 to 13.1)	1.08 (0.96 to 1.22)	1.00	
	TSG-h	13.3 (12.4 to 14.3)	12.5 (11.7 to 13.5)	1.06 (0.94 to 1.20)	1.00	
	TSG-l	13.0 (12.2 to 13.9)	11.8 (10.9 to 12.7)	1.11 (0.97 to 1.26)	0.47	
HGS (kg)	CG	56.0 (53.1 to 59.0)	56.8 (53.8 to 59.9)	0.99 (0.94 to 1.03)	1.00	CG/SSSG-h = 1.00; CG/SSSG-l = 1.00; CG/TSG-h = 1.00; CG/TSG-l = 1.00; SSSG-h/SSSG-l = 1.00; SSSG-h/TSG-h = 1.00; SSSG-h/TSG-l = 1.00; SSSG-l/TSG-h = 1.00; SSSG-l/TSG-l = 1.00; TSG-h/TSG-l = 1.00;
	SSSG-h	57.8 (53.5 to 62.5)	60.4 (55.8 to 65.3)	0.96 (0.89 to 1.02)	1.00	
	SSSG-l	57.6 (53.6 to 61.8)	59.0 (54.8 to 63.6)	0.98 (0.91 to 1.04)	1.00	
	TSG-h	59.5 (54.9 to 64.4)	62.0 (57.2 to 67.2)	0.96 (0.89 to 1.02)	1.00	
	TSG-l	53.1 (49.3 to 57.3)	54.3 (50.1 to 58.8)	0.98 (0.91 to 1.05)	1.00	
HJ (cm)	CG	128 (122 to 134)	128 (122 to 135)	0.99 (0.92 to 1.08)	1.00	CG/SSSG-h = 0.53; CG/SSSG-l = 1.00; CG/TSG-h = 1.00; CG/TSG-l = 1.00; SSSG-h/SSSG-l = 1.00; SSSG-h/TSG-h = 0.54; SSSG-h/TSG-l = 1.00; SSSG-l/TSG-h = 1.00; SSSG-l/TSG-l = 1.00; TSG-h/TSG-l = 1.00
	SSSG-h	128 (121 to 136)	139 (130 to 148)	0.92 (0.83 to 1.02)	0.55	
	SSSG-l	128 (121 to 136)	132 (124 to 141)	0.97 (0.88 to 1.08)	1.00	
	TSG-h	128 (120 to 137)	127 (119 to 136)	1.01 (0.91 to 1.12)	1.00	
	TSG-l	126 (119 to 134)	134 (125 to 144)	0.94 (0.84 to 1.05)	1.00	

CG, Control group; SSSG-h, small-sided soccer games group with high attendance (over 50% attendance); SSSG-l, small-sided soccer games group with attendance low attendance (below 50% attendance); TSG-h, Traditional soccer group with high attendance (over 50% attendance); TSG-l, Traditional soccer group with low attendance (below 50% attendance); HGS, handgrip strength; HJ, horizontal jump

Data: Estimated mean (confidence interval 95%). Models adjusted for baseline values of the dependent variable and peak height velocity.
Ratios represent baseline-to-post comparisons (Pre/Post) of estimated marginal means derived from the generalized linear mixed model with gamma distribution and log link. Estimates are back-transformed from the log scale. Ratios <1 indicate a reduction from baseline to post-intervention, whereas ratios >1 indicate an increase.

Supplementary table 5. Results by attendance at exercise sessions for cardiometabolic risk factors

	Group	Baseline	Post-intervention	pre/post ratio (IC95%)	p-value	p-value between-group
Heart rate (bpm)	CG	76.87 (73.55 to 80.34)	75.37 (71.83 to 79.07)	1.02 (0.94 to 1.11)	1.00	CG/SSSG-h = 0.14; CG/SSSG-l = 1.00; CG/TSG-h = 0.61; CG/TSG-l = 1.00; SSSG-h/SSSG-l = 0.57; SSSG-h/TSG-h = 1.00; SSSG-h/TSG-l = 0.07; SSSG-l/TSG-h = 1.00; SSSG-l/TSG-l = 1.00; TSG-h/TSG-l = 0.26;
	SSSG-h	77.68 (72.80 to 82.88)	68.45 (64.15 to 73.04)	1.14 (1.01 to 1.27)	**0.01**	
	SSSG-l	76.69 (72.18 to 81.48)	74.34 (69.47 to 79.55)	1.03 (0.92 to 1.16)	1.00	
	TSG-h	76.83 (71.85 to 82.16)	69.47 (64.96 to 74.29)	1.11 (0.99 to 1.24)	0.17	
	TSG-l	75.56 (70.89 to 80.53)	76.65 (71.00 to 82.74)	0.99 (0.87 to 1.12)	1.00	
PAS (mmHg)	CG	110.51 (107.36 to 113.76)	105.03 (101.77 to 108.41)	1.05 (0.99 to 1.11)	0.18	CG/SSSG-h = 1.00; CG/SSSG-l = 1.00; CG/TSG-h = 0.73; CG/TSG-l = 1.00; SSSG-h/SSSG-l = 1.00; SSSG-h/TSG-h = 1.00; SSSG-h/TSG-l = 1.00; SSSG-l/TSG-h = 1.00; SSSG-l/TSG-l = 1.00; TSG-h/TSG-l = 1.00;
	SSSG-h	111.44 (106.78 to 116.31)	105.90 (101.48 to 110.52)	1.05 (0.97 to 1.14)	1.00	
	SSSG-l	110.94 (106.64 to 115.41)	109.15 (104.41 to 114.12)	1.02 (0.94 to 1.10)	1.00	
	TSG-h	111.51 (106.83 to 116.40)	111.81 (107.12 to 116.72)	0.99 (0.92 to 1.08)	1.00	
	TSG-l	110.61 (106.24 to 115.16)	109.10 (103.92 to 114.53)	1.01 (0.93 to 1.10)	1.00	
PAD (mmHg)	CG	66.51 (64.19 to 68.92)	64.13 (61.70 to 66.65)	1.04 (0.97 to 1.11)	1.00	CG/SSSG-h = 0.47; CG/SSSG-l = 1.00; CG/TSG-h = 1.00; CG/TSG-l = 1.00; SSSG-h/SSSG-l = 0.11; SSSG-h/TSG-h = 0.33; SSSG-h/TSG-l = 1.00; SSSG-l/TSG-h = 1.00; SSSG-l/TSG-l = 0.94; TSG-h/TSG-l = 1.00;
	SSSG-h	66.96 (63.58 to 70.52)	60.16 (57.12 to 63.37)	1.11 (1.01 to 1.22)	**0.008**	
	SSSG-l	66.20 (63.05 to 69.50)	66.05 (62.53 to 69.77)	1.00 (0.91 to 1.10)	1.00	
	TSG-h	66.78 (63.34 to 70.41)	65.41 (62.04 to 68.96)	1.02 (0.93 to 1.12)	1.00	
	TSG-l	67.22 (63.95 to 70.64)	62.39 (58.78 to 66.22)	1.08 (0.97 to 1.19)	0.79	
Insuline	CG	14.89 (12.51 to 17.72)	13.81 (11.53 to 16.54)	1.08 (0.77 to 1.510)	1.00	CG/SSSG-h = 0.41; CG/SSSG-l = 1.00; CG/TSG-h = 1.00; **CG/TSG-l = 0.04**; SSSG-h/SSSG-l = 0.17; SSSG-h/TSG-h = 0.32; **SSSG-h/TSG-l = < 0.001**; SSSG-l/TSG-h = 1.00; SSSG-l/TSG-l = 0.76; TSG-h/TSG-l = 0.25.
	SSSG-h	15.78 (12.25 to 20.31)	10.28 (7.99 to 13.23)	1.53 (0.97 to 2.417)	0.09	
	SSSG-l	14.31 (11.44 to 17.89)	15.34 (11.57 to 20.34)	0.93 (0.56 to 1.554)	1.00	
	TSG-h	15.00 (11.75 to 19.14)	14.89 (11.67 to 19.01)	1.01 (0.64 to 1.582)	1.00	
	TSG-l	13.26 (10.53 to 16.70)	21.01 (15.77 to 27.97)	0.63 (0.38 to 1.048)	0.14	
HOMA-IR	CG	3.29 (2.72 to 3.97)	3.13 (2.57 to 3.81)	1.05 (0.73 to 1.514)	1.00	CG/SSSG-h = 0.42; CG/SSSG-l = 1.00; CG/TSG-h = 1.00; **CG/TSG-l = 0.04**; SSSG-h/SSSG-l = 0.21; SSSG-h/TSG-h = 0.45; SSSG-h/TSG-l = **< 0.001**; SSSG-l/TSG-h = 1.00; SSSG-l/TSG-l = 0.65; TSG-h/TSG-l = 0.19;
	SSSG-h	3.56 (2.71 to 4.68)	2.31 (1.76 to 3.04)	1.54 (0.94 to 2.530)	0.21	
	SSSG-l	3.13 (2.46 to 3.99)	3.43 (2.53 to 4.66)	0.91 (0.52 to 1.594)	1.00	
	TSG-h	3.38 (2.59 to 4.40)	3.37 (2.59 to 4.39)	1.00 (0.61 to 1.640)	1.00	
	TSG-l	2.88 (2.24 to 3.70)	4.90 (3.59 to 6.70)	0.59 (0.34 to 1.023)	0.08	
HbA1c (%)	CG	5.01 (4.97 to 5.06)	5.07 (5.02 to 5.12)	0.99 (0.97 to 1.009)	1.00	CG/SSSG-h = 1.00; CG/SSSG-l = 1.00; CG/TSG-h = 0.23; CG/TSG-l = 0.09; SSSG-h/SSSG-l = 1.00; SSSG-h/TSG-h = 0.39; SSSG-h/TSG-l = 0.18; SSSG-l/TSG-h = 1.00; SSSG-l/TSG-l = 1.00; TSG-h/TSG-l = 1.00;
	SSSG-h	5.03 (4.96 to 5.10)	5.08 (5.02 to 5.15)	0.99 (0.96 to 1.016)	1.00	
	SSSG-l	5.02 (4.96 to 5.08)	5.15 (5.08 to 5.23)	0.97 (0.95 to 1.002)	0.12	
	TSG-h	5.03 (4.96 to 5.10)	5.20 (5.13 to 5.27)	0.97 (0.94 to 0.992)	**0.001**	
	TSG-l	5.02 (4.95 to 5.08)	5.21 (5.13 to 5.29)	0.96 (0.94 to 0.990)	**<0.001**	
Glucose (mg/dl)	CG	89.18 (86.94 to 91.47)	91.34 (88.97 to 93.76)	0.98 (0.93 to 1.03)	1.00	CG/SSSG-h = 1.00; CG/SSSG-l = 1.00; CG/TSG-h = 1.00; CG/TSG-l = 1.00; SSSG-h/SSSG-l = 1.00; SSSG-h/TSG-h = 1.00; SSSG-h/TSG-l = 0.07; SSSG-l/TSG-h = 1.00; SSSG-l/TSG-l = 0.56; TSG-h/TSG-l = 1.00;
	SSSG-h	90.18 (87.01 to 93.46)	88.90 (85.78 to 92.14)	1.01 (0.95 to 1.08)	1.00	
	SSSG-l	89.07 (86.22 to 92.02)	89.66 (86.18 to 93.28)	0.99 (0.93 to 1.07)	1.00	
	TSG-h	89.48 (86.36 to 92.71)	91.62 (88.43 to 94.94)	0.98 (0.92 to 1.04)	1.00	
	TSG-l	88.18 (85.26 to 91.20)	94.17 (90.42 to 98.08)	0.94 (0.87 to 1.01)	0.12	
TC (mg/dl)	CG	151.96 (145.97 to 158.19)	134.82 (129.35 to 140.52)	1.13 (1.05 to 1.21)	**<0.0001**	CG/SSSG-h = 1.00; CG/SSSG-l = 1.00; CG/TSG-h = 0.07; CG/TSG-l = 1.00; SSSG-h/SSSG-l = 1.00; SSSG-h/TSG-h = 0.51; SSSG-h/TSG-l = 1.00; **SSSG-l/TSG-h = 0.02**; SSSG-l/TSG-l = 1.00; TSG-h/TSG-l = 1.00;
	SSSG-h	148.13 (140.04 to 156.70)	133.65 (126.33 to 141.38)	1.11 (1.01 to 1.21)	**0.008**	
	SSSG-l	151.70 (143.95 to 159.86)	130.22 (122.53 to 138.39)	1.17 (1.05 to 1.29)	**<.0001**	
	TSG-h	148.70 (140.52 to 157.35)	144.76 (136.79 to 153.19)	1.03 (0.94 to 1.12)	1.00	
	TSG-l	150.88 (143.08 to 159.10)	138.11 (129.69 to 147.09)	1.09 (0.99 to 1.21)	0.17	
HDL (mg/dl)	CG	45.25 (43.11 to 47.50)	42.41 (40.36 to 44.57)	1.07 (0.99 to 1.15)	0.29	CG/SSSG-h = 1.00; CG/SSSG-l = 0.42; CG/TSG-h = 0.33; CG/TSG-l = 1.00; SSSG-h/SSSG-l = 0.22; SSSG-h/TSG-h = 0.17; SSSG-h/TSG-l = 0.82; SSSG-l/TSG-h = 1.00; SSSG-l/TSG-l = 1.00; TSG-h/TSG-l = 1.00;
	SSSG-h	45.98 (43.04 to 49.12)	42.15 (39.45 to 45.03)	1.09 (0.98 to 1.21)	0.28	
	SSSG-l	44.26 (41.57 to 47.11)	45.23 (42.04 to 48.65)	0.98 (0.87 to 1.10)	1.00	
	TSG-h	45.53 (42.55 to 48.73)	46.46 (43.41 to 49.71)	0.98 (0.88 to 1.09)	1.00	
	TSG-l	45.07 (42.33 to 47.98)	44.86 (41.68 to 48.29)	1.01 (0.89 to 1.13)	1.00	
LDL (mg/dl)	CG	84.52 (78.77 to 90.69)	73.97 (68.81 to 79.51)	1.14 (1.03 to 1.27)	**0.001**	CG/SSSG-h = 1.00; CG/SSSG-l = 1.00; CG/TSG-h = 0.82; CG/TSG-l = 1.00; SSSG-h/SSSG-l = 0.30; SSSG-h/TSG-h = 1.00; SSSG-h/TSG-l = 1.00; **SSSG-l/TSG-h = 0.05**; SSSG-l/TSG-l = 1.00; TSG-h/TSG-l = 0.82;
	SSSG-h	79.94 (72.45 to 88.21)	73.85 (66.92 to 81.50)	1.08 (0.94 to 1.25)	1.00	
	SSSG-l	83.57 (76.20 to 91.66)	67.15 (60.45 to 74.58)	1.25 (1.06 to 1.46)	**<.0001**	
	TSG-h	80.37 (72.78 to 88.75)	77.23 (69.92 to 85.31)	1.04 (0.90 to 1.20)	1.00	
	TSG-l	81.04 (73.96 to 88.81)	69.63 (62.58 to 77.47)	1.16 (0.99 to 1.36)	0.07	

VLDL (mg/dl)	CG	19.49 (16.88 to 22.49)	15.73 (13.53 to 18.29)	1.24 (0.97 to 1.58)	0.20	CG/SSSG-h = 1.00; CG/SSSG-l = 1.00; CG/TSG-h = 1.00; CG/TSG-l = 0.64; SSSG-h/SSSG-l = 1.00; SSSG-h/TSG-h = 0.77; SSSG-h/TSG-l = 0.19; SSSG-l/TSG-h = 1.00; SSSG-l/TSG-l = 0.42; TSG-h/TSG-l = 1.00;
	SSSG-h	19.39 (15.90 to 23.66)	14.16 (11.59 to 17.30)	1.37 (0.99 to 1.89)	0.06	
	SSSG-l	20.40 (16.94 to 24.56)	15.39 (12.39 to 19.11)	1.33 (0.93 to 1.89)	0.44	
	TSG-h	18.50 (15.12 to 22.63)	17.28 (14.11 to 21.17)	1.07 (0.78 to 1.48)	1.00	
	TSG-l	19.61 (16.10 to 23.90)	20.22 (16.10 to 25.39)	0.97 (0.68 to 1.38)	1.00	
Triglyceride (mg/dl)	CG	96.86 (83.94 to 111.77)	78.97 (67.95 to 91.77)	1.23 (0.96 to 1.57)	0.29	CG/SSSG-h = 1.00; CG/SSSG-l = 1.00; CG/TSG-h = 1.00; CG/TSG-l = 0.73; SSSG-h/SSSG-l = 1.00; SSSG-h/TSG-h = 1.00; SSSG-h/TSG-l = 0.25; SSSG-l/TSG-h = 1.00; SSSG-l/TSG-l = 0.34; TSG-h/TSG-l = 1.00;
	SSSG-h	96.89 (79.46 to 118.14)	71.95 (58.92 to 87.84)	1.35 (0.98 to 1.86)	0.11	
	SSSG-l	101.98 (84.74 to 122.72)	76.03 (61.24 to 94.40)	1.34 (0.94 to 1.91)	0.32	
	TSG-h	91.99 (75.24 to 112.47)	85.59 (69.91 to 104.80)	1.08 (0.78 to 1.48)	1.00	
	TSG-l	97.47 (79.99 to 118.76)	100.64 (80.17 to 126.34)	0.97 (0.68 to 1.38)	1.00	
SPISE	CG	5.47 (5.21 to 5.75)	5.85 (5.56 to 6.16)	1.07 (0.89 to 1.28)	0.29	CG/SSSG-h = 1.00; CG/SSSG-l = 1.00; CG/TSG-h = 1.00; CG/TSG-l = 0.65; SSSG-h/SSSG-l = 1.00; SSSG-h/TSG-h = 1.00; SSSG-h/TSG-l = 0.19; SSSG-l/TSG-h = 0.84; **SSSG-l/TSG-l = 0.05;** TSG-h/TSG-l = 1.00;
	SSSG-h	5.48 (5.12 to 5.86)	6.05 (5.65 to 6.48)	0.95 (0.74 to 1.21)	0.09	
	SSSG-l	5.45 (5.12 to 5.80)	6.19 (5.75 to 6.67)	1.17 (0.89 to 1.52)	0.01	
	TSG-h	5.37 (5.01 to 5.75)	5.62 (5.24 to 6.02)	0.85 (0.67 to 1.08)	1.00	
	TSG-l	5.49 (5.15 to 5.85)	5.42 (5.02 to 5.84)	1.04 (0.80 to 1.36)	1.00	
AST/ALT ratio	CG	1.14 (1.04 to 1.25)	1.06 (0.97 to 1.17)	1.29 (1.04 to 1.61)	1.00	CG/SSSG-h = 1.00; CG/SSSG-l = 1.00; CG/TSG-h = 0.13; CG/TSG-l = 1.00; SSSG-h/SSSG-l = 0.56; SSSG-h/TSG-h = 1.00; SSSG-h/TSG-l = 1.00; **SSSG-l/TSG-h = 0.04;** SSSG-l/TSG-l = 1.00; TSG-h/TSG-l = 0.64;
	SSSG-h	1.11 (0.98 to 1.26)	1.17 (1.03 to 1.34)	1.27 (0.95 to 1.71)	1.00	
	SSSG-l	1.23 (1.09 to 1.39)	1.05 (0.91 to 1.22)	1.29 (0.94 to 1.77)	1.00	
	TSG-h	1.10 (0.97 to 1.26)	1.30 (1.14 to 1.48)	1.18 (0.88 to 1.59)	1.00	
	TSG-l	1.21 (1.07 to 1.37)	1.16 (1.00 to 1.35)	1.31 (0.95 to 1.81)	1.00	
GGT (U/L)	CG	20.77 (18.71 to 23.04)	16.06 (14.40 to 17.91)	1.07 (0.89 to 1.28)	**0.006**	CG/SSSG-h = 1.00; CG/SSSG-l = 1.00; CG/TSG-h = 1.00; CG/TSG-l = 1.00; SSSG-h/SSSG-l = 1.00; SSSG-h/TSG-h = 1.00; SSSG-h/TSG-l = 1.00; SSSG-l/TSG-h = 1.00; SSSG-l/TSG-l = 1.00; TSG-h/TSG-l = 1.00;
	SSSG-h	21.53 (18.65 to 24.85)	16.94 (14.65 to 19.59)	0.95 (0.74 to 1.21)	0.36	
	SSSG-l	20.88 (18.26 to 23.88)	16.19 (13.73 to 19.08)	1.17 (0.89 to 1.52)	0.40	
	TSG-h	19.15 (16.52 to 22.20)	16.18 (13.95 to 18.76)	0.85 (0.67 to 1.08)	1.00	
	TSG-l	21.02 (18.31 to 24.13)	16.03 (13.51 to 19.01)	1.04 (0.80 to 1.36)	0.28	

CG, Control group; SSSG-h, small-sided soccer games group with high attendance (over 50% attendance); SSSG-l, small-sided soccer games group with attendance low attendance (below 50% attendance); TSG-h, Traditional soccer group with high attendance (over 50% attendance); TSG-l, Traditional soccer group with low attendance (below 50% attendance); SBP, systolic blood pressure; DBP, diastolic blood pressure; HOMA-IR, homeostatic model assessment for insulin resistance; HbA1c, glycated hemoglobin A1c, TC, total cholesterol; HDL-C, high-density lipoprotein cholesterol; LDL-C, low-density lipoprotein cholesterol; VLDL-C, very low-density lipoprotein cholesterol; TG, triglycerides; ALT, alanine aminotransferase; AST, aspartate aminotransferase; GGT, γ-glutamyl transpeptidase; SPISE, single-point insulin sensitivity estimator

Data: Estimated mean (confidence interval 95%). Models adjusted for baseline values of the dependent variable and peak height velocity.
